# Chemoautotrophy in subzero environments and the potential for cold-adapted Rubisco

**DOI:** 10.1128/aem.00604-25

**Published:** 2025-05-30

**Authors:** Kaitlin Harrison, Josephine Z. Rapp, Alexander L. Jaffe, Jody W. Deming, Jodi Young

**Affiliations:** 1 School of Oceanography, University of Washington 189579, Seattle, Washington, USA; 2 Astrobiology Program, University of Washington, Seattle, Washington, USA; 3 REV Ocean 683293 , Fornebu, Norway; 4 Department of Earth System Science, Stanford University 539510, Stanford, California, USA; University of Milano-Bicocca, Milan, Italy

**Keywords:** Rubisco, astrobiology, chemoautotrophy, autotrophs, psychrophiles

## Abstract

The act of fixing inorganic carbon into the biosphere is largely facilitated by one enzyme, Rubisco. Beyond well-studied plants and cyanobacteria, many bacteria use Rubisco for chemolithoautotrophy in extreme environments on Earth. Here, we characterized the diversity of autotrophic pathways and chemolithoautotrophic Rubiscos from two distinct subzero, hypersaline Arctic environments: 40-kyr relic marine brines encased within permafrost (cryopeg brines) and first-year sea ice. The Calvin-Benson-Bassham (CBB) cycle was widely found in both environments, although with different predominant Rubisco forms. From cryopeg brine, reconstructions of metagenome-assembled genomes (MAGs) uncovered four MAGs with the potential for chemolithoautotrophy, of which the CBB-containing genus *Thiomicrorhabdus* was most abundant. A broader survey of *Thiomicrorhabdus* genomes from diverse environments identified a core complement of three Rubisco forms (II, IAc, IAq) with a complex pattern of gain and loss, with form II constitutively present in genomes from subzero environments. Using representative kinetic data, we modeled carboxylation rates of Rubisco forms II, IAc, and IAq across CO_2_, O_2_, and temperature conditions. We found that form II outcompetes form I at low O_2_, but cold temperatures minimize this advantage. Inspection of form II from genomes from cold environments identified signals of potential thermal adaptation due to key amino acid substitutions, which resulted in a more exposed active site. We argue that subzero form II from *Thiomicrorhabdus* warrants further study as it may have unique kinetics or thermal stability. This work can help address the limits of autotrophic functionality in extreme environments on Earth and other planetary bodies.

**IMPORTANCE:**

Autotrophy, or the fixation of inorganic carbon to biomass, is a key factor in life’s ability to thrive on Earth. Research on autotrophy has focused on plants and algae, but many bacteria are also autotrophic and can survive and thrive under more extreme conditions. These bacteria are a window to past autotrophy on Earth, as well as potential autotrophy in extreme environments elsewhere in the universe. Our study focused on dark, cold, saline environments, which are likely to be found on Enceladus and Europa, as well as in the Martian subsurface. We found evidence for potential cold adaptation in a key autotrophic enzyme, Rubisco, which could expand the known boundaries of autotrophy in rapidly disappearing icy environments on Earth. We also present a novel model framework that can be used to probe the limits of autotrophy not only on Earth but also on key astrobiological targets like Enceladus and Europa.

## INTRODUCTION

Autotrophy, or the fixation of inorganic carbon to complex organic molecules, is required for life in almost every ecosystem on Earth. In addition, the earliest known life on Earth is often believed to be autotrophic (1–3). While recent work on protein evolution has cast doubt on our understanding of the physiology of the last common ancestor (4), what is certain is the early emergence of autotrophy in the history of life on Earth and its importance in a thriving Earth biosphere.

Seven carbon fixation pathways occur naturally on Earth (5, 6). However, estimates of global carbon fixation suggest that the Calvin-Benson-Bassham (CBB) cycle is responsible for >99% of the autotrophy on the planet (7). This cycle is the only carbon fixation pathway in oxygenic photosynthesis and thus is found in every plant, cyanobacterium, and alga. It is also utilized in chemolithoautotrophs, coupled to a diverse set of bacterial metabolisms including oxidation of sulfur, iron, ammonia, and nitrite (7). While likely not the earliest carbon fixation pathway to evolve (3), it is found in almost every habitat on the planet regardless of temperature, pressure, salinity, and oxygen concentration. Due to its prevalence, enzyme diversity in the CBB cycle is much better sampled than in any of the other autotrophic carbon fixation pathways, making it an incredibly useful tool to study the adaptation of carbon fixation to various environmental conditions.

The maximum speed of the CBB cycle is typically controlled by the enzyme ribulose 1,5-bisphosphate carboxylase/oxygenase [EC 4.1.1.39], also known as Rubisco. In the CBB cycle, Rubisco catalyzes the step in which CO_2_ is added to a five-carbon organic sugar (ribulose 1,5-bisphosphate, or Rubp) to form two 3-phosphoglycerate molecules. Thus, Rubisco is directly responsible for the conversion of inorganic carbon to organic carbon. Despite its critical role in the CBB cycle, Rubisco is not well adapted to our current atmospheric CO_2_ and O_2_ concentrations (8). Rubisco is competitively inhibited by oxygen, resulting in the production of 2-phosphoglycolate (2-PG), a toxic molecule whose removal via a phosphoglycolate salvage (PGS) pathway results in the loss of recently fixed carbon. In plants, up to 49% of gross primary production can be lost via PGS (also called photorespiration) (9). During the Archean, when Rubisco evolved (10), this side reaction would have been rare due to the relatively high CO_2_ levels and extremely low O_2_ levels. However, as O_2_ levels have risen and CO_2_ levels have dropped, PGS has become a significant source of carbon loss. Rubisco is also poorly suited for the temperatures at which it typically operates across most of Earth’s surface. *In vitro* assays demonstrate that Rubisco has a maximum activity between 45°C and 60°C (11), temperatures that most of the ocean and land never reach. At Earth’s average surface temperature (15°C), Rubisco has somewhere between 4% and 13% of its peak activity (11).

There are four known forms of Rubisco (12). Form I Rubisco is a hexadecamer, made of eight large subunits and eight small subunits. Form I can be further split into subtypes (forms IA–IE), with forms IB and ID found in eukaryotic autotrophs and some cyanobacteria, and forms IA, IC, and IE exclusively found in bacteria. Form II Rubisco, which is only composed of large subunits, can form a wide range of oligomers depending on species; it is found in bacteria and some algae (dinoflagellates). Form III Rubisco and form IV Rubisco are thought to be involved in other processes such as nucleoside salvage rather than autotrophic carbon fixation (13–16), although there are exceptions (5). Recently discovered clades, including I’, Iα, and II/III, are beginning to challenge our notion of distinct forms (17, 18). While all eukaryotic algae and plants only contain one form of Rubisco, many proteobacteria that utilize the CBB cycle often encode multiple forms of Rubisco (19), and multiple forms of Rubisco were recently found in a cyanobacterium from marine oxygen minimum zones (20).

Despite decades of Rubisco research, there remain outstanding questions about why this tremendous amount of enzyme diversity exists. One hypothesis is that diversity is driven by the environmental pressures of the aforementioned mismatch between optimal conditions for Rubisco activity and the current conditions on Earth. The ability of Rubisco to adapt to a particular environment continues to be debated due to its extremely slow rate of evolution (21). However, the available data are dominated overwhelmingly by plant Rubisco form IB (11) measured at a canonical 25°C (22) and thus lack power for testing environmental adaptation. Many non-IB forms of Rubisco have kinetics that vary significantly from form IB (23, 24). Most studies have focused on a suspected trade-off between carboxylation speed and affinities for CO_2_ over O_2_ (23, 24). An increase in speed is advantageous under high CO_2_ (8), and many organisms have evolved an intracellular carbon-concentrating mechanism to elevate CO_2_ around Rubisco (25). One such carbon-concentrating mechanism is the carboxysome, found in cyanobacteria and some chemolithoautotrophic bacteria. This bacterial protein structure excludes O_2_ while allowing CO_2_ to be concentrated around Rubisco (25).

Considerably less is known about the role of temperature in driving Rubisco diversity. Like most enzymes, Rubisco carboxylation rate (*k*_cat,C_) increases with temperature. Increasing temperature also decreases the CO_2_ specificity (S_C/O_) and the CO_2_ affinity (K_C_) (26). There is still debate as to whether cold adaptation exists in Rubisco. Minor differences in temperature sensitivity have been observed between plants typically found in cold versus warm environments (26). Few measurements of temperature dependence have been made outside the form IB Rubiscos (26). Even fewer are from extremophiles, with most measurements from thermophiles rather than psychrophiles (26). To date, there is a lack of compelling experimental data for cold adaptation of Rubisco. While form ID from a psychrophilic alga (a diatom) may perform slightly better at cold temperatures than mesophilic diatoms (27), this needs to be confirmed with purified Rubisco. Form ID Rubiscos from cold-temperature seaweeds have slightly higher *k*_cat,C_ at cold temperatures than their mesophilic counterparts (28), but their Rubiscos have not yet been sequenced. To date, no study has been conducted on Rubisco from psychrophilic bacteria.

Subzero environments are extreme environments facing significant changes in the next decades due to climate change. The Arctic is predicted to be ice free in the summer by the 2030s (29), and the infiltration of permafrost melt into homes and food storage is a potential hazard to residents of the North, especially Indigenous communities (30). Taxon-specific traits are needed to model the polar carbon fixation response to these changes (31). Beyond Earth, the 2020 Astronomy Decadal Survey has called for more research on poly-extreme environments as analogs for similar environments on exoplanets (32). Permafrost brines and sea ice are particularly analogous to conditions on the icy moons of Jupiter and Saturn, especially Europa and Enceladus, which are considered high-priority targets in the search for life in our solar system (32).

A survey of literature on chemoautotrophic taxa in subzero environments, including high-latitude seawater in winter, terrestrial brines, endolithic habitats, polar brines, and sea ice, revealed diverse autotrophic taxa that, combined, covered five of the seven known natural carbon fixation pathways (Table 1). The CBB cycle was present in every subzero environment where chemoautotrophy was detected, regardless of temperature or oxygen concentration. The prevalence of chemoautotrophic pathways suggests chemoautotrophy may play a larger role in subzero environments than previously supposed. One genus of chemolithoautotrophic bacteria, *Thiomicrorhabdus*, was detected in four different high-salinity habitats across both poles. This genus of Gammaproteobacteria has been confirmed to be actively growing in subzero brines in the Canadian Arctic (33).

**TABLE 1:** Survey of potential chemoautotrophy in subzero environments^*a*^

Environment	Autotrophic pathway	Known chemoautotrophs^*b*^	Temperature (°C)^*c*^	Ref.
Seawater				
Winter surface seawater, Western Antarctic Peninsula	CBB	Gammaproteobacteria (Candidatus Ruthia magnifica)	Avg. –1.73	(34)
Water under Ross Sea ice shelf, Antarctica	CBB, 3-HP, 3-HP/4-HB, rTCA	Gammaproteobacteria (SUP05, UBA10353), Alphaproteobacteria, Nitrososphaeria (*Nitrosopumilus*), Nitrospiria (*Nitrospira*)	Avg. –2	(35)
Brine				
Cryopeg brines, Utqiaġvik, Alaska	CBB	Gammaproteobacteria (*Thiomicrorhabdus, Methylophaga*), Actinobacteria (*Aeromicrobium*)	–6 to –8	This study
Blood Falls brine, Antarctica	CBB	Gammaproteobacteria (*Thiomicrorhabdus*), Chloroflexi	–7.8 (36)	(37)
Lost Hammer Spring, Axel Heiberg, Canada	CBB, W-L, rTCA	Gammaproteobacteria (*Thiomicrorhabdus*), Alphaproteobacteria, Actinobacteriota, Desulfurobacterota, Campylobacterota	–5	(38)
Gypsum Hill Spring, Axel Heiberg, Canada	CBB, rTCA	Gammaproteobacteria (*Halothiobacillus, Thiomicrorhabdus*), Epsilonproteobacteria (*Sulfuricurvum, Sulfurimonas*)	–1.3 to 6.9	(39, 40)
Lake Vida brine, Antarctica	CBB	Gammaproteobacteria (*Thiomicrorhabdus*)	–13	(41)
Other				
Blood Falls rocks, Antarctica	CBB, W-L, rTCA	Candidatus Dormibacterota, Eremiobacteriota, Actinobacteriota, Chloroflexota, Gemmatimonadota, Proteobacteria	Variable, but in summer varies diurnally between –10 and 10 (42)	(43)

^*a*^ CBB, Calvin-Benson-Bassham cycle; W-L, Wood-Ljungdahl pathway; rTCA, reductive TCA cycle; 3-HP, 3-hydroxyproprionate bi-cycle; 3-HP/4-HB, 3-hydroxyproprionate/4-hydroxybutyrate cycle.

^*b*^ If autotrophy was mentioned, autotrophic members were categorized and noted. If autotrophy was not mentioned, community members from genera or families known to be obligate autotrophs were considered to be autotrophic and listed with their known autotrophic pathway. Only chemolithoautotrophs were included for this analysis.

^*c*^ If available, temperature information from the same study was used. If not, temperature was collected from other studies of the same habitat.

To further investigate chemoautotrophy at subzero temperatures, we first quantified the abundance of key marker genes for chemoautotrophic pathways in metagenomes from two distinct subzero environments: ancient marine brines within the permafrost (cryopeg brines) and first-year sea ice. We then quantified the abundance of chemoautotrophic Rubisco forms (IA, IC, IE, and II) in these metagenomes. We further explored Rubisco diversity in *Thiomicrorhabdus*, an obligate autotroph that uses the CBB cycle to fix carbon (44), due to its prevalence in subzero environments and multiple Rubisco forms. We also developed a generalizable theoretical carboxylation model to examine the trade-off between Rubisco forms in *Thiomicrorhabdus* under different CO_2_, O_2_, and temperature regimes. Finally, we examined genetic sequences of Rubiscos from cold-inhabiting *Thiomicrorhabdus* for signals of thermal adaptation.

## RESULTS

### Autotrophy within cryopeg brines and sea ice

We probed available metagenomic data sets for chemoautotrophy within the subzero environments of first-year Arctic sea-ice brines (–3.5°C and 77 ppt) and cores (–4°C and 18 ppt), and stable, anoxic relic-marine brines trapped within permafrost (cryopeg brines, –6°C and 123 ppt). Samples were collected from Utqiaġvik in 2017 and 2018 (for full description of sampling, see references 45 and 46). Potential chemoautotrophic metabolisms were detected within all metagenomes sampled from cryopeg and sea-ice samples, with bacterial communities in cryopeg brines possessing a higher abundance of autotrophic-associated genes than in sea ice (45).

**Fig 1:**
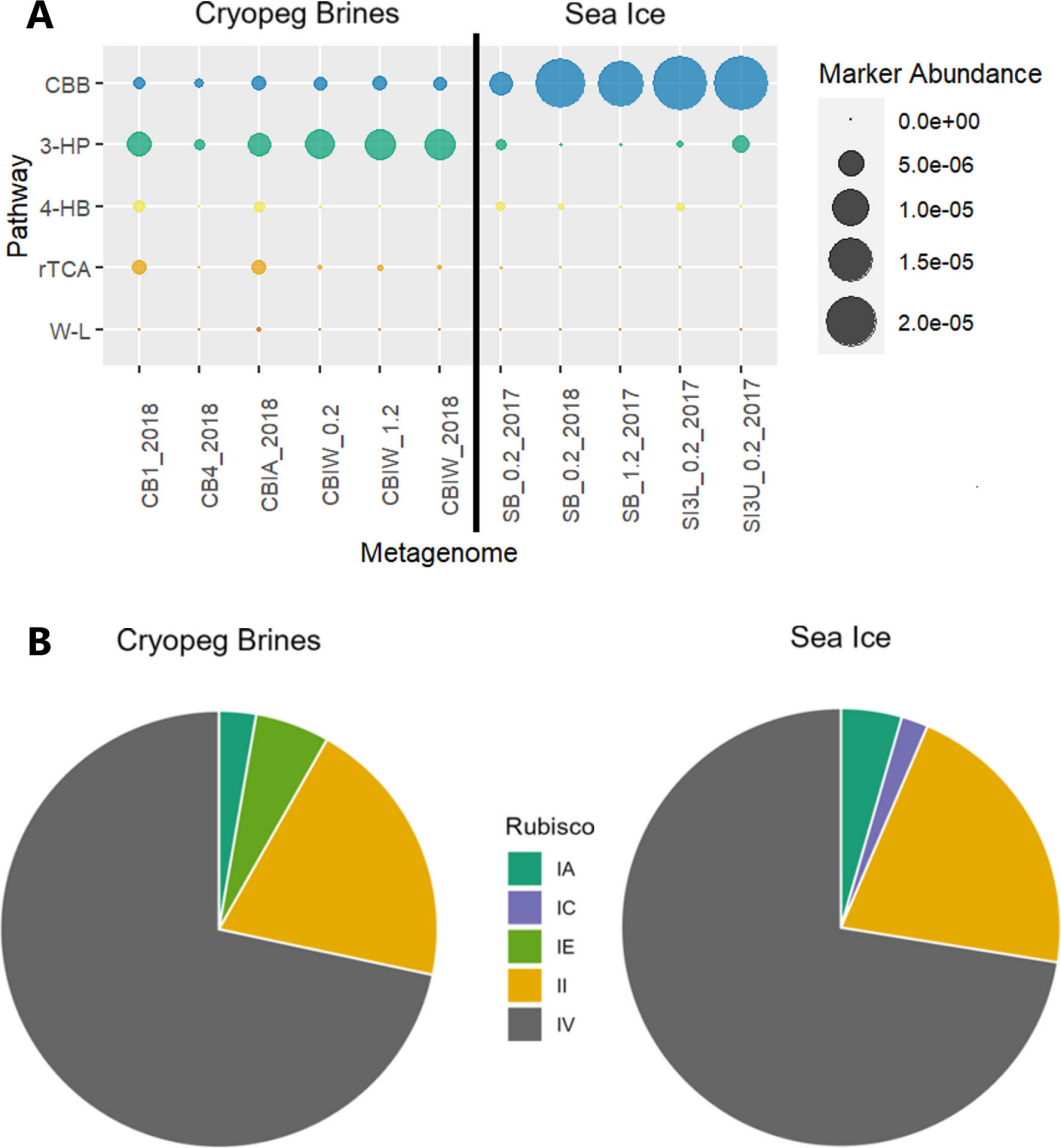
Rubisco forms in metagenomes from cryopeg brines and sea ice. (**A**) Relative abundance of key genes from carbon fixation pathways. Abundance calculated from mapped reads to gene-encoding scaffolds (see Table S1 for the list of genes used). CB metagenomes from cryopeg brine; SB metagenomes from sea-ice brine; SI metagenomes from melted sea ice. See Reference 45 for metagenome details. Abbreviations: 4-HB, 4-hydroxybutyrate/dicarboxylic acid cycle and 3-hydroxyproprionate/4-hydroxybutyrate cycle; others shared with Table 1. (**B**) Proportion of bacterial Rubisco abundance attributed to each form within cryopeg brine and sea-ice metagenomes. Abundance calculated from mapped reads to Rubisco-encoding scaffolds from bacteria only. No form III was detected in either cryopeg brine or sea-ice samples. The number of sequences and the proportion of read depth attributed to form I subforms and form II in Fig. S4.

Using the same metagenomic data sets, we used three approaches to identify potential chemoautotrophic pathways: the abundance of marker genes, the presence of metagenome-assembled genomes (MAGs) with a complete autotrophic pathway, and a further dive into one marker gene with known forms used in heterotrophy versus autotrophy. First, we identified which potential autotrophic pathways were present (Fig. 1A) based on key genes (Table S1). The CBB cycle is highly represented in sea ice, with the other pathways present in low abundances. In cryopeg brines, the most abundant marker gene was 2-methylfumaryl-CoA isomerase [E.C. 5.4.1.3], the key gene of the 3-HP bicycle. The CBB cycle was also present in all cryopeg brine samples, typically at lower abundances than the 3-HP bicycle (one- to sixfold lower). The other pathways were detected primarily in two metagenomes (CB1_2018 and CBIA_2018) and were either absent (W-L) or in very low abundances in the other cryopeg brine samples (rTCA, 4-HB/DC and 3-HP/4-HB).

Although marker gene abundance provides a basic indication of the abundance of metabolic pathways, it may overestimate the presence of the pathway due to either an incomplete pathway existing in the organism or its use in a different pathway. Thus, we also explored chemoautotrophic pathways by looking for full pathways in MAGs assembled from the cryopeg brine and sea-ice metagenomes. No MAGs with a full chemoautotrophic pathway were found in the sea-ice metagenomes, but five were found in the cryopeg brine metagenomes.

Three of the MAGs belonged to Gammaproteobacteria (two different *Thiomicrorhabdus* MAGs, hereafter called UC1 and UC2, and a *Methylophaga* MAG) and one to Actinobacteria (*Aeromicrobium*), all with a full CBB cycle (Table 2). The fifth MAG was identified as belonging to the genus *Sulfurospirillum* and encoded the rTCA cycle. As the genus *Sulfurospirillum* has not been shown to grow autotrophically, despite the presence of the full rTCA cycle (47, 48), it was not considered autotrophic going forward. Full details of MAG completeness and contamination are shown in Table S2. The contribution of putative autotrophic members to the total cryopeg brine community was small, with 0.5%–1.5% of the total reads mapping to the autotrophic MAGs across all samples (Fig. S1).

**TABLE 2:** Cryopeg brine MAG completeness and Rubisco complement

MAG name/identity	Completeness (%)	Contamination (%)	Rubisco forms present^*a*^		
			Form II	Form IAq	Form IE
UC1/*Thiomicrorhabdus arctica*	97.42	0.00	Y	Y	N
UC2/*Thiomicrorhabdus* spp.	98.37	0.61	Y	N	N
*Aeromicrobium*	94.34	4.00	N	N	Y^*b*^
*Methylophaga*	99.65	1.07	Y	N	N

^*a*^ Y, yes; N, no.

^*b*^ Only the RbcL subunit is present.

Average nucleotide identities (ANIs) of the cryopeg *Thiomicrorhabdus* MAGs UC1 and UC2 (Fig. S2) identified UC1 as a new strain of *Thiomicrorhabdus arctica* (98.62% ANI), while UC2 was most likely a new species, with the closest match to *Thiomicrorhabdus* sp. NP51 (89.79% ANI), a strain of *Thiomicrorhabdus* isolated from a Canadian cold saline spring (33).

The UC1 genome contained both form II and form IA Rubisco, consistent with the previously isolated and sequenced strain of *T. arctica* (44). UC2 contained only form II Rubisco. The neighboring genes and their order around the form II cassette remained the same as those surrounding the form II and form IA cassette in *T. arctica*, the closest cultured relative of UC2 (Fig. S3), leading us to conclude that this was a true loss of form IA from UC2.

To address the possibility of marker genes being used in other pathways, we subsequently took a closer look at the CBB marker gene (Rubisco), which has taxon-specific forms that indicate its use in autotrophy versus heterotrophy. A total of 774 redundant sequences attributed to the Rubisco large subunit were detected across all samples, with the vast majority (693 sequences or 89.5%) derived from sea ice. Most of the major forms of Rubisco (forms I–IV) were detected in both cryopeg brine and sea-ice samples, with the notable exception of form III. None of the recently discovered (sub)forms (i.e., I’, Iα, II/III) were detected. Many Rubisco sequences from sea-ice samples were associated with algae (forms IB and ID). As photosynthetic eukaryotes are not the target of this study, these algal sequences were not analyzed further.

Bacterial Rubiscos were mostly attributed to Gamma- and Alphaproteobacteria in both cryopeg brines and sea ice. The non-autotrophic form IV was the most abundant (Fig. 1B; 76% and 72% of mapped reads for cryopeg brines and sea ice, respectively). Chemoautotrophic Rubiscos were found across all samples except for one cryopeg brine sample, which had a different bacterial community and geochemistry (46), and one sea-ice sample, in which only photoautotrophic Rubisco sequences were found. Form II was most abundant, followed by IA and IE in cryopeg brines or IA and IC in sea ice. Half of the form II and all of the form IA Rubisco sequences in the cryopeg brines were attributed to *Thiomicrorhabdus*.

### Rubisco in *Thiomicrorhabdus*

Species from the genus *Thiomicrorhabdus* are obligate chemoautotrophs (44). Its presence in our cryopeg brines and known autotrophic activity in subzero environments (33, 37) led us to focus further on this genus and its Rubisco diversity.

There are 45 publicly available *Thiomicrorhabdus* genomes (MAGs and cultured isolates) from a variety of habitats that encompass a wide range of temperature (and salinity), with cultured strains able to grow from –2°C to 45°C (and 0 to 1,700 mM NaCl or approximately 0 to 99 ppt) (Table S2). Two of these genomes were sampled from subzero environments, and an additional seven cultured isolates grow below 5°C. Known sampling locations are displayed in Fig. S5. Where available, the temperature range and other information (salinity range, quality, source) for each genome are given in Table S2.

**Fig 2:**
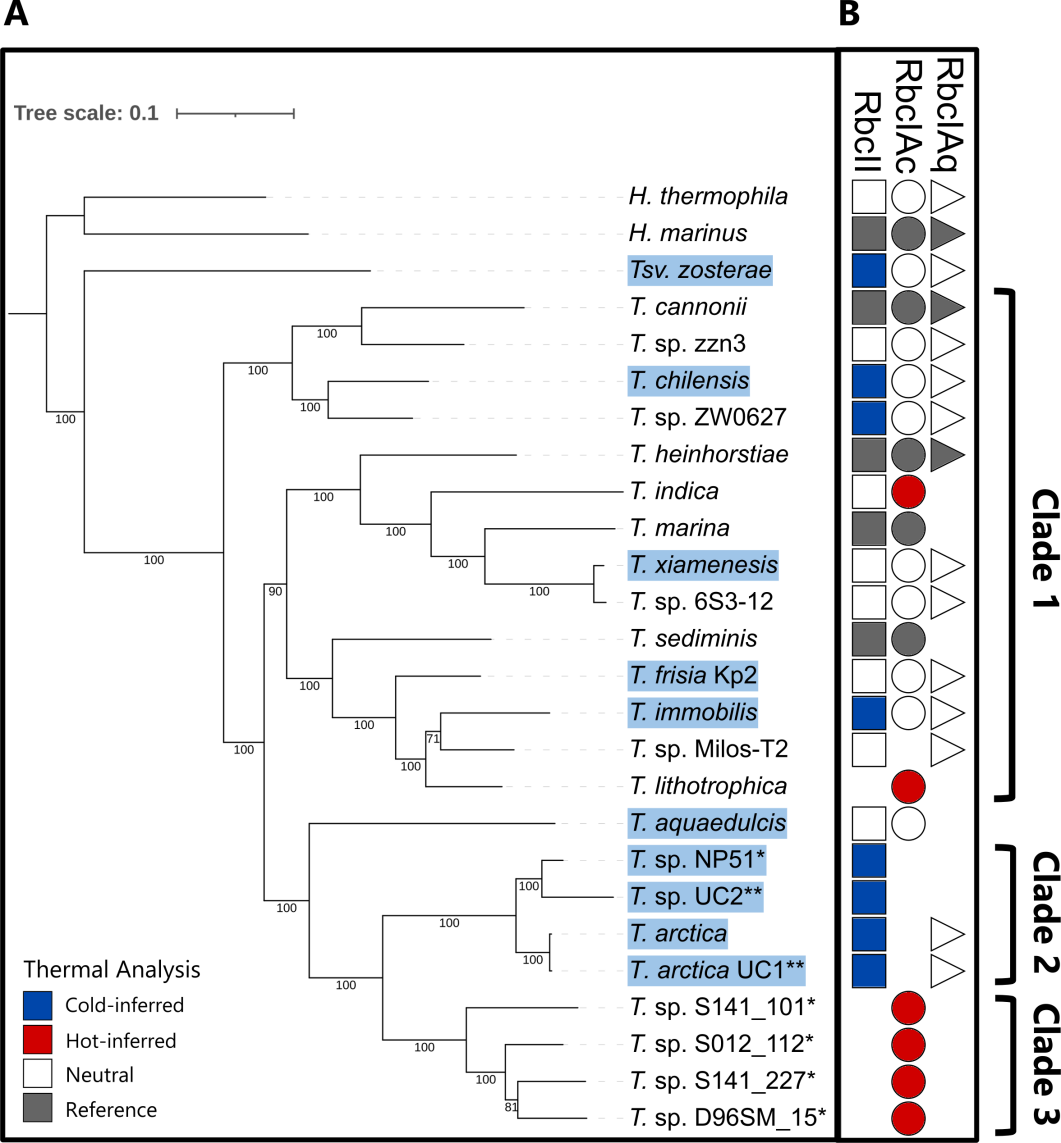
Phylogenetic tree of *Thiomicrorhabdus* genomes alongside Rubisco gene complement and results from amino acid thermal analysis. (**A**) Maximum-likelihood phylogenetic tree of 22 *Thiomicrorhabdus* genomes. *H. marinus* and *H. thermophila* designated as outgroup. Bootstrap values for each node are depicted on the branch. MAGs designated with (*). Genomes added in this study designated with (**). Highlighting in blue shows species found in stable cold environments or with reported growth at ≤5°C. *Thiosulfativibrio (Tsv.) zosterae* reclassified after inclusion. (**B**) Presence/absence of Rubisco forms and result of thermal adaptation analysis. Shape represents the presence of form: square, form II; circle, form IAc; triangle, form IAq. Color indicates the result of thermal analysis: blue, cold-inferred; red, hot-inferred; outline only, neutral; gray, used as reference for thermal analysis. See Fig. S6 for an expanded Clade 3 that includes two other genomes in Clade 3 that passed the quality control but did not contain a complete Rubisco gene.

We compiled a data set of 17 cultured genomes and 6 MAGs that passed quality control (<5% contamination, >75% completeness, and de-replication) for further analysis. A maximum likelihood phylogenetic tree was constructed from these *Thiomicrorhabdus* genomes, plus an outgroup of two genomes from a closely related genus, *Hydrogenovibrio* (Fig. 2A). Our cryopeg MAGs UC1 and UC2 and other genomes isolated from subzero environments formed a distinct clade (Clade 2) based on ANI. All *Thiomicrorhabdus* genomes encoded at least one Rubisco gene, with many encoding up to three different forms: form II and two forms of IA, one cytosolic (IAq), and the other a carboxysome-associated form (IAc) (Fig. 2B). Both form II and form IAq were found in a cassette with their associated transcriptional regulator LysR gene and Rubisco activase (CbbQ/CbbO dimer pair) (Fig. S7). Form IAc was surrounded by carboxysome genes, as previously observed (49). Rubisco gene duplication was only detected in *Thiomicrorhabdus sediminis*, which contained two copies of form IAc. Both *Hydrogenovibrio* genomes encoded all three forms of Rubisco found in the *Thiomicrorhabdus* genomes.

The distribution pattern of Rubisco forms within *Thiomicrorhabdus* is complex and likely cannot be explained by a few instances of gene loss or gain. Interestingly, all genomes isolated from subzero environments encoded form II but not form IAc (Fig. 2, Clade 2), whereas a clade of genomes from hydrothermal vents only encoded form IAc (Fig. 2, Clade 3). We acknowledge that the absence of a gene is difficult to confirm in a MAG; however, five of the MAGs are ≥90% complete, including all of the Clade 2 MAGs and two of the Clade 3 MAGs, and the same complex distribution of Rubisco genes remained when only considering genomes from cultured isolates. For example, within Clade 1, cultured isolates *Thiomicrorhabdus immobilis* (previously Am19), *Thiomicrorhabdus* sp. Milos-T2, and *Thiomicrorhabdus lithotrophica* (previously XGS-01) are all closely related based on marker genes but encode different complements of Rubisco genes.

### Theoretical framework to compare Rubisco kinetics under diverse environmental conditions

It has been hypothesized that multiple Rubisco forms within proteobacteria are advantageous under different environmental conditions (19, 49). To test this hypothesis in *Thiomicrorhabdus*, we derived a theoretical framework and compared different Rubisco forms within a matrix of CO_2_, O_2_, and temperature conditions. As kinetics data from *Thiomicrorhabdus* are not available, we used the published kinetics data of the three Rubisco forms (form II, form IAq, and form IAc) from closely related *Hydrogenovibrio marinus (*50, 51). Rubisco kinetics for each form (carboxylation speed, *k*_cat,C_; half-saturation constants for CO_2_, K_C_, and O_2_, K_O_; and CO_2_/O_2_ specificity, S_C/O_) along with estimated temperature effects (26) were used to model Rubisco carboxylation across CO_2_ and O_2_ concentrations at two temperatures (0°C and 25°C; see Methods and Tables S3 to S6). The influence of temperature is uncertain due to limited data, resulting in both form IAs having similar temperature dependence equations (Fig. S8). Temperature dependence differences were more apparent between form IA and form II, such that form II *k*_cat,C_ increases more rapidly than form IA as temperature increases, while form II S_C/O_ decreases less sharply than form IA as temperature increases (Fig. S8). For full details, see Methods and Tables S3 to S6.

**Fig 3:**
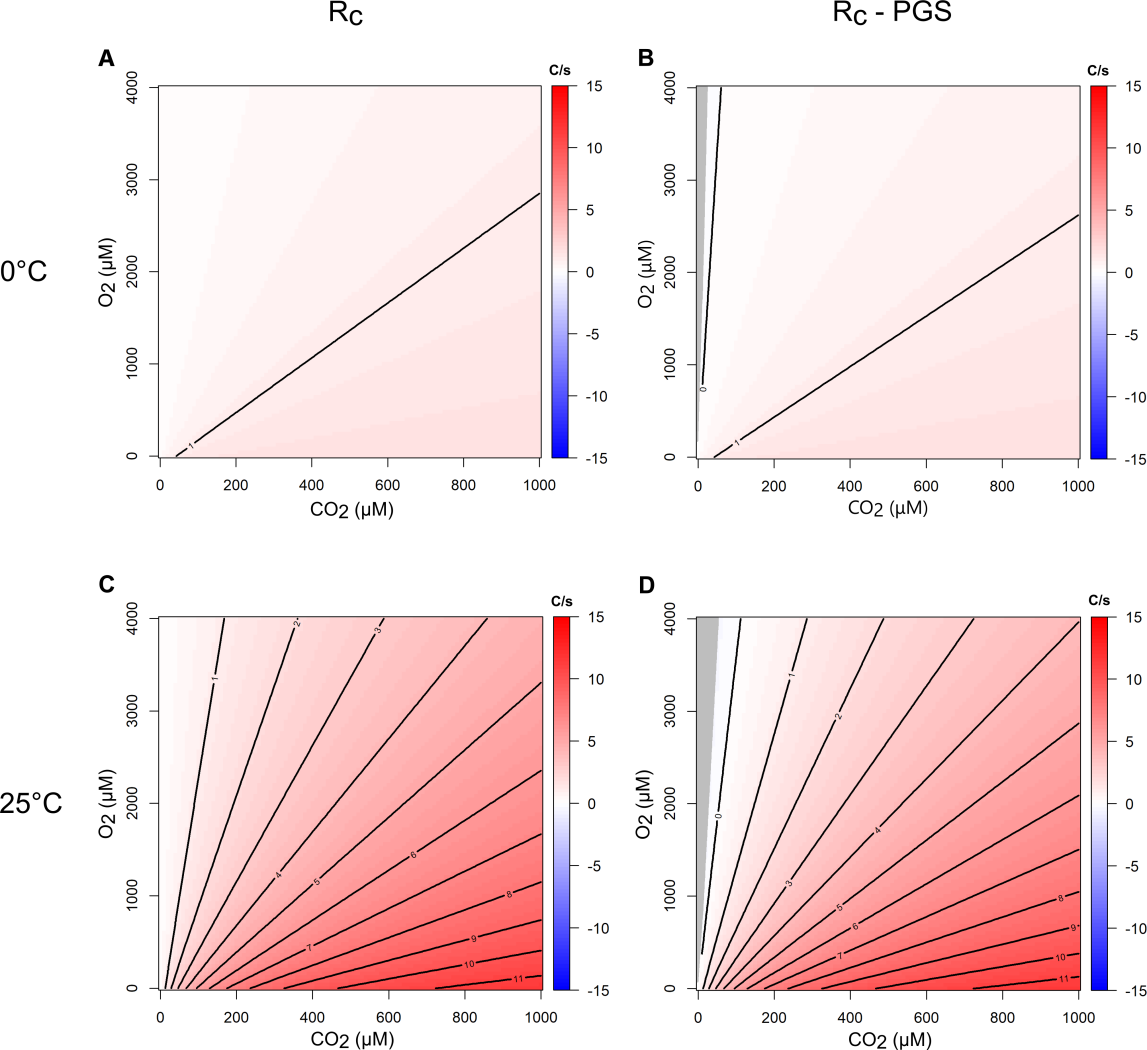
Theoretical kinetics model of Rubisco net carboxylation. Modeled Rubisco carboxylation speed for forms II–IAc at varying CO_2_ concentration, O_2_ concentration, and temperature. Red indicates form II > IAc, blue indicates form IAc > II, with shading indicating the magnitude in carbons per second (C/s). Gray shading at low CO_2_ indicates conditions where there was a net loss of carbon for both enzymes. (A) and (C) show gross carboxylation rate equation (R_C_); (B) and (D) show carboxylation rate when the PGS pathway (C2 cycle) is included at 0°C and 25°C, respectively. All graphs share a color scale.

The differences in carboxylation rates between Rubisco forms across the CO_2_/O_2_ parameter space at the three temperatures are shown in Fig. 3. Rates were also modeled including the loss of CO_2_ due to PGS (Fig. 3; Table S6). To determine which PGS pathway to use, *Thiomicrorhabdus* genomes were searched for key enzymes from the four known PGS pathways (52). No *Thiomicrorhabdus* genome contained the key genes for the malate cycle, the glycerate pathway, or the oxalyl-CoA decarboxylation route. Key enzymes for the C2 cycle were present in most or all of the *Thiomicrorhabdus* genomes, so we modeled *Thiomicrorhabdus* enzymes using the C2 cycle stoichiometry.

The rate of Rubisco carboxylation of form II was always faster than forms IAc and IAq (Fig. 3A and C). However, incorporation of the PGS pathway resulted in form IAc outcompeting form II under warm, oxygenated conditions with low CO_2_ (Fig. 3B and D). The form IAq enzyme was outcompeted by IAc and II under all conditions.

Decreasing temperature reduced the difference in carboxylation speed between forms IAc and II. At 0°C, the two enzymes had at most a 2 carbon-per-second (C/s) difference (Fig. 3A and B). At 25°C, form II was up to 11 C/s faster than IAc (Fig. 3C and D), although form IAc outcompeted form II over a larger CO_2_/O_2_ parameter space.

### Thermal adaptation analysis

Many cold-adapted enzymes display several amino acid substitutions that enhance kinetics at colder temperatures (53). While no one substitution is evidence of thermal adaptation, surveying a range of these indices against a mesophilic control can be used to infer whether thermal adaptation is likely. To assess the potential for thermal adaptation for each Rubisco sequence, we used the approach of Raymond-Bouchard et al. (53) and surveyed 10 different amino acid substitutions (indices) commonly associated with thermal adaptation against their mesophilic average (comprised of mesophilic *Thiomicrorhabdus* Rubiscos of the same form; Table S7). In short, each index received a score of +1 if significantly cold-adapted compared to the mesophilic average and –1 if significantly hot-adapted. Scores over all 10 indices were summed for a final signal. We considered a sequence to contain a signal of cold or hot adaptation (hereafter called a cold-inferred or hot-inferred sequence) after the arbitrary cutoff values of >3 or <–3, respectively, as in previous studies (see Methods for details).

**Fig 4:**
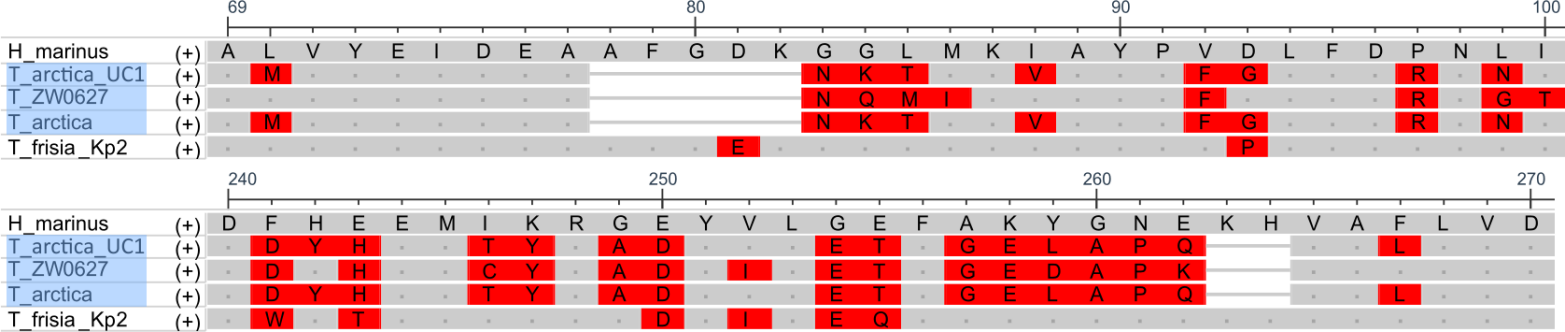
Partial amino acid alignments of Rubisco form II. Rubisco form II sequence excerpts from *H. marinus*, *T. frisia* Kp2, *T. arctica*, *T. arctica* UC1, and *T*. sp. ZW0627 around common, prominent substitutions/gaps found within the cold-inferred sequences (blue) compared to the neutral *H. marinus* and *T. frisia* Kp2. Amino acids highlighted in red show deviation from the *H. marinus* sequence.

Of the 44 Rubisco sequences present in the genomes, 19 (43%) displayed a signal of thermal adaptation (Fig. 2B). All of the cold-inferred sequences were Rubisco form II sequences, including all of the Clade 2 form IIs (those from subzero environments). Other cold-inferred form II sequences were found in cultured isolates that were able to grow at ≤5°C (Fig. 2; Table S2), with the exception of one in a cultured isolate (*T*. sp. ZW0627) with no known temperature range. All cold-inferred form IIs had similar amino acid substitutions and gaps (Fig. 4; Fig. S9). The cold-inferred Rubisco form II sequences had a median score of +4, with the majority of the 10 indices (5–7) scoring as significantly cold-adapted in all but one sequence (*Thiosulfativibrio zosterae*). Form II in the type strain of *T. arctica* had the highest number of cold-adapted indices of the group, with 7 of the 10 indices pointing toward cold adaptation (Table S8).

All hot-inferred Rubisco sequences were form IAc. In particular, genomes with only form IAc all showed a signal of hot adaptation in that gene, including every MAG in Clade 3, which were all sampled from hydrothermal vent fields. In general, the scores for thermal adaptation in form IAc were weaker than those for form II (Table S8). No form IAq sequence was cold- or hot-inferred.

### Rubisco form II structural analysis

**Fig 5:**
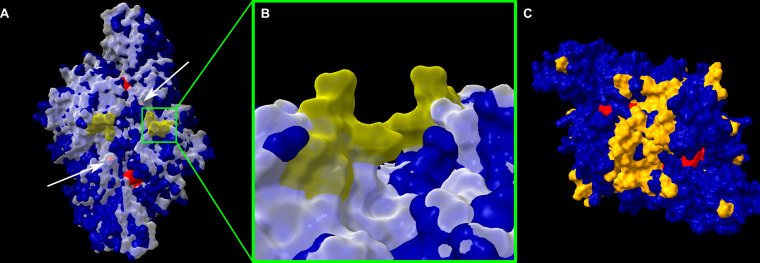
Rubisco form II structural differences between *T. arctica* UC1 and *H. marinus*. (**A**) AlphaFold-generated structures for *H. marinus* (transparent white) and *T. arctica* UC1 (dark blue) Rubisco form II dimers. Active sites are colored red. Arrows point to the additional exposed active site residues in UC1 compared to *H. marinus*. The missing residues in UC1 at amino acid positions 78–82 (Fig. 4) are colored yellow on *H. marinus* structure (a close-up view in B). (**C**) UC1 form II dimer with residue differences to *H. marinus* that confer a semi-conservative or non-conservative change highlighted in orange.

To investigate potential structural differences in form II Rubisco sequences with and without a signal of cold adaptation, three cold-inferred (*T.* sp. NP51, *Thiomicrorhabdus chilensis*, and *T. arctica* UC1) and three neutral (*Thiomicrorhabdus indica*, *Thiomicrorhabdus frisia* Kp2, and *Thiomicrorhabdus aquaedulcis*) sequences were compared to a reference form II from *H. marinus*. Proteins were created as dimers using AlphaFold (54, 55). All of the cold-inferred structures had significant differences to the *H. marinus* structure that the neutral structures did not contain (Fig. 5; Fig. S10).

Many amino acid substitutions or gaps in the cold-inferred form II sequences changed the surface of the protein, primarily between the two active sites (Fig. 5C). One set of deletions present in the cold-inferred form II structures corresponded to an absence of residues that protrude from the surface of the protein between the active sites in the *H. marinus* and neutral structures (Fig. 5B; amino acids 78–82). The other deletions are residues that form a small loop on the back of the protein in the neutral structures (Fig. S10B). In addition to the deletions, the active site was more exposed in cold-inferred form II structures because of two substitutions (Y304P and P312A, Fig. 5A arrows), although the active site residues themselves remained unchanged (Fig. S10A). These differences were not present in the neutral structures (Fig. S10E through G).

## DISCUSSION

### Chemoautotrophic diversity in subzero environments

In our two subzero environments, key genes for all of the known autotrophic pathways were found (with the exception of the reductive glycine pathway, which cannot be distinguished from the oxidative version of the pathway and thus was not explored in this study). The 3-HP bicycle, the most abundant pathway in cryopeg brines, is an oxygen-tolerant pathway that has only recently been found outside of Chloroflexaceae (56), which are anoxygenic photosynthesizers and therefore not likely to be found in the permanently dark cryopeg brines. The high abundance of the 3-HP bicycle in these brines and yet the absence of a complete pathway in the cryopeg brine MAGs may indicate its use as an organic carbon incorporation pathway, as seen in *Chloroflexi aurantiacus* (57) and hypothesized for other MAGs with incomplete pathways (56). Surprisingly, autotrophic pathways common in anoxic environments (W-L and the rTCA cycle) were not predominant pathways even in the anoxic cryopeg brines. The archaeal 4-HB cycles (4-HB/DC and 3-HP/4-HB) were also not found in abundance in accordance with the lack of archaea in our metagenomes.

The abundance of the CBB cycle in sea ice is unsurprising given the prevalence of sea-ice algae in these environments, but its presence in cryopeg brines was surprising. While many of the bacteria with Rubisco use the non-autotrophic form IV Rubisco for other reasons, we found a diverse range of chemoautotrophic bacterial Rubisco forms within Arctic cryopeg brines and sea-ice samples (forms II, IA, and IE or IC, respectively). Forms II, IA, and IC are commonly found in proteobacteria, which formed a high percentage of the community in both environments (46). The absence of form IC from cryopeg brines could be a taxonomic signal due to a population bottleneck after cryopeg formation (58). The original population of the cryopeg brines may have contained proteobacteria with form IC, which were lost by chance during the rapid population drop. However, it is also possible that these proteobacteria were simply less well suited to the environment of the cryopeg brines, which are colder and saltier than sea-ice brines, and anoxic. Its counterpart, form IE, present in cryopeg brines but not in sea-ice samples, may reflect the terrestrial influence of the surrounding permafrost on the cryopeg brines. Form IE is associated with soil and atmospheric Actinobacteria (12) and is the predominant form of Rubisco in some polar deserts (59). All of the form IE sequences in our study were attributed to *Actinobacteria*, and many of them, including our cryopeg brine MAG with form IE, are from the family *Nocardiaceae*, which has been previously found in permafrost soils (60, 61).

Form II was several times more abundant than form IA in both our cryopeg brines and sea-ice samples. This finding could be a taxonomic signal if form II is simply more common than form IA in all proteobacteria. Conversely, it could be a signal of environmental selection, as form II is typically faster than form I under low O_2_ (50) such as in cryopeg brines. In oxygenated sea ice, the prevalence of form II may be due to the increased solubility of CO_2_ versus O_2_ at low temperatures.

### *Thiomicrorhabdus* as a model organism to study multiple Rubisco forms

The prevalence of *Thiomicrorhabdus* in subzero environments presented a unique opportunity to study Rubisco form and function across extreme environments. Not only is this genus active across diverse subzero environments (Lost Hammer Spring and Gypsum Hill Spring in the Canadian Arctic—see references 33 and 38; and in the outflow brine from Blood Falls in Antarctica—see reference 37), it is also found near hydrothermal vents, in coastal sediments, and in briny lakes (44). While cultured strains have been reported to grow from –2°C to 45°C, they have been detected in environments with temperatures as low as –13°C (41).

The majority of *Thiomicrorhabdus* genomes have three different forms of Rubisco (II, IAc, IAq), similar to the *Hydrogenovibrio* outgroup, suggesting that the common *Thiomicrorhabdus* ancestor contained all three forms. Our model output of form II versus form IA based on *Hydrogenovibrio* Rubisco kinetics found that form IAc outperforms form II at 25°C only at a high ratio of O_2_/CO_2_. We also found no environmental space where form IAq would be faster at fixing carbon than form IAc or form II. These results are consistent with laboratory studies of *H. marinus* that found form II preferentially transcribed under high CO_2_ (62), form IAc expressed with its carboxysome under low CO_2_, and form IAq expressed solely as a supplemental form under high and low but not median CO_2_. *H. marinus* form II Rubisco is the third-fastest form II measured to date but has a low specificity for CO_2_ (50); thus, our model output may be different when using form II kinetics from other bacteria.

Why chemoautotrophic bacteria but not oxygenic photoautotrophs have retained multiple copies of Rubisco is not known. Badger and Bek hypothesized that environments with fluctuating geochemistry or microenvironments, where many chemoautotrophs reside, favor retention of multiple Rubisco forms (19). Recently, a cyanobacterium inhabiting anoxic marine waters was shown to express both form I and form II Rubisco (20), suggesting that oxygen may at least in part influence the acquisition (and expression) of isoforms. As *Thiomicrorhabdus* species are known to inhabit environments across a wide range of O_2_ (0–0.6 mM; see references 33, 46, 63 and 64) and CO_2_ conditions (2.3–7.1 mM; see reference 49), the diversity of Rubisco isoforms in *Thiomicrorhabdus* may provide an evolutionary benefit, allowing species to use a Rubisco isoform tailored to the ambient conditions while still maintaining flexibility for rapid changes in conditions as might occur in sediment microenvironments or hydrothermal vent gradients.

The complex pattern of loss of Rubisco forms in *Thiomicrorhabdus* suggests either that there is some cost to maintaining multiple copies of Rubisco (e.g., genome streamlining; see reference 65) or a specialization of some *Thiomicrorhabdus* species to a narrower range of conditions. With the diversity of habitats and gene complements for *Thiomicrorhabdus* species, this genus provides an excellent opportunity for future work on multiple forms of Rubisco in a single organism.

### Rubisco adaptation to extreme environments

As temperatures decrease, the variability in Rubisco kinetics collapses for most parameters (excluding S_C/O_; see reference 26). Our model also reproduces this observation, as the competitive advantage of form II is reduced at 0°C compared to 25°C. Our model was dependent on extrapolating kinetic properties from a limited data set, which was particularly sparse for temperature sensitivity. However, even a small increase in Rubisco net carboxylation may provide a competitive advantage for the cell, particularly when accounting for the high abundance of Rubisco at cold temperatures (27). The prevalence of form II in subzero environments and our finding of a potential cold-adaptation signal within our cold-tolerant form II protein sequences suggest that form II may be capable of thermal adaptation that would increase its carboxylation at cold temperatures.

Most studies have found little evidence that form I Rubisco in C3 plants from cool climates (66) or from psychrophilic algae (67, 68) has a faster *k*_cat,C_ or lower Q_10_ (temperature coefficient). The few exceptions include experiments on whole cell extracts from some polar seaweeds (28) and a diatom (69) with a slightly higher *k*_cat,C_ than their temperate counterparts, although the Q_10_ is still within the known range for plants. Rubiscos from thermophiles have a higher temperature optimum of activity (70–73), but little is known about this parameter in psychrophiles. Cold adaptation in Rubisco may also improve Rubisco assembly and activation rather than affecting kinetics directly, as assembly and activation are highly complex at cold temperatures (27).

Like other Rubiscos (21), all forms of Rubisco within the Piscirickettsiaceae family (of which *Thiomicrorhabdus* is a member) are under purifying selection (74). However, even a few amino acid substitutions can have a significant effect on Rubisco kinetics (21, 75), and a number of studies allude to co-evolution of Rubisco kinetics and their intracellular environment (21). Our AlphaFold modeling found a more exposed active site in our cold Rubiscos, and efforts are underway to determine whether these structural changes confer shifts in Rubisco activity, assembly, or kinetics compared to their mesophilic counterparts.

Our study did not focus on Rubisco forms at high temperatures. Nonetheless, we did observe the importance of form IAc in hydrothermal vent MAGs, which also displayed a signal for high-temperature adaptation in the protein sequence. Form IAc is associated with a carboxysome, which would be advantageous at high temperatures where CO_2_ solubility is low.

This study and others (62) found no evidence for a need to retain form IAq within genomes under the conditions tested (CO_2_, O_2_, temperature). However, synthesizing a carboxysome requires a high amount of nutrient investment to build the protein shell, and we speculate that form IAq may be useful when organisms are nutrient limited rather than energy limited.

### Comparing different Rubisco forms by modeling kinetics

We developed a modeling framework to compare carboxylation rates per active site for different Rubisco forms under environmental conditions beyond the canonical atmospheric conditions at 25°C so often quoted. This approach allowed us to explore the environmental limits of autotrophy and could be used to identify viable environmental conditions for autotrophy on other worlds (e.g., in Martian subsurface brines or the Enceladus ocean).

This framework can also explore the use of Rubisco in organisms with multiple forms of Rubisco. We propose that in organisms with multiple Rubiscos, a form is more likely to be preferentially expressed under conditions where it performs better than the other available forms. The agreement between our model and laboratory studies of *H. marinus* validates this approach, while underlining a need for further experiments that compare the model predictions to Rubisco expression patterns in *Thiomicrorhabdus*. A unique component of our model is the consideration of the Rubisco oxygenase reaction through incorporation of the carbon loss due to PGS pathways. Only when a PGS pathway is added to the model is there a parameter space where form IA outcompetes form II, and also where Rubisco carboxylation is no longer a viable strategy for autotrophy.

Outside of *Thiomicrorhabdus*, this same modeling approach can be applied to other Rubisco proteins with known kinetics in other species and to approximate specific environments of interest. We found that under low oxygen conditions, form II is likely to be more common, a finding strengthened by the recent detection of a cyanobacterial ecotype from marine oxygen-deficient zones with an additional form II Rubisco (20). Form II may thus be a better analog model for carbon fixation on the icy moons Enceladus or Europa, which are thought to be anoxic (76). In contrast, Earth’s current atmospheric conditions of high O_2_ and low CO_2_ (for geologic time) result in both forms II and IA losing carbon; in these cases, a carboxysome might be utilized to further exclude oxygen and increase CO_2_ around Rubisco. The current CO_2_ and O_2_ regime also supports the dominance of form IB in the modern Earth atmosphere, as form IB in plants has a higher S_C/O_ than form IA (10).

### Conclusion

Overall, our study has provided multiple lines of evidence that form II Rubisco has a competitive advantage at cold temperatures. First, it is the most abundant autotrophic bacterial Rubisco form found in our metagenomes from subzero cryopeg brines and sea ice. Second, it is the only form present in all *Thiomicrorhabdus* genomes from cold environments. Third, our model indicates it performs better than Rubisco form I over the majority of conditions at cold temperatures. Fourth, many of the form II Rubisco amino acid sequences contained a signal of cold adaptation with potential structural changes on the surface of the protein.

Our study also shows that the use of models that incorporate PGS, and the exploration of multiple forms of Rubisco within a single genus, can provide valuable information to probe the environmental limits at which Rubisco and the CBB cycle can operate. Ultimately, this work underscores the need for more data on Rubisco kinetics from non-plant organisms, especially chemolithoautotrophic bacteria, and the importance of studying autotrophy in extreme environments for understanding current life on Earth and potential life on other planetary bodies.

## MATERIALS AND METHODS

### Identification of autotrophy marker genes from cryopeg brines and sea ice

Genes were identified from metagenomes from cryopeg and sea-ice sackhole brines and sea-ice top and bottom core melts in May 2017 and May 2018, sampled as described in Rapp et al. (45). Briefly, sea ice from ~1.1 to 1.2 m thick first-year land-fast sea ice was sampled ~100 m from the Barrow Sea Ice Mass Balance site (71.37219°N, 156.53828°W) in early spring. Sea-ice brines were collected from sackholes, and sea-ice core melts (from the top third and bottom third of the core) were collected from the same ice field. Cryopeg brines were sampled from the Barrow Permafrost Tunnel (71.2944°N, 156.7153°W), 6 m below the surface. Brines were extracted from approximately 2 m below the tunnel floor using a hand pump and sterile receiving system. Sample nomenclature and environmental data are in Table S9. Further site and sampling details can be found in references 45 and 46.

From these samples, a set of 11 metagenomes was previously generated and described by reference 45. Briefly, sequencing was performed at the DOE Joint Genome Institute (JGI) on an Illumina NovaSeq sequencer, and data from individual samples were subsequently quality controlled, assembled, and structurally and functionally annotated through the DOE–JGI Metagenome Annotation Pipeline (MAP v4.16) (77). For the analyses of metabolic pathways and pathway completion assessment, we focused on KEGG Orthology (KO) term assignments. Through MAP, genes were associated with KO terms based on the results of a sequence similarity search of metagenome proteins against the IMG-NR reference database v20181114 using lastal 914 from the LAST package (78), with default parameters. Coverage information was obtained by mapping filtered reads back to assembled contigs using BBMap v38.25 with “ambiguous = random.” Gene taxonomy was inferred through a homology search using LAST (78) against IMG reference isolates (high-quality public genomes), and scaffold lineage affiliation then represents the last common ancestor of all best gene hits on a scaffold, provided that at least 30% of the genes had hits.

### Rubisco form assignment

All nucleotide sequences annotated as the Rubisco large subunit were translated into protein sequences, with any sequences less than 100 amino acids discarded. Amino acid sequences were aligned using MAFFT (79) with 66 reference sequences encompassing different Rubisco forms from Prywes et al. (12). A maximum likelihood tree was built using IQTREE2 (80), and Rubisco form was assigned based on the closest reference sequences.

Forms ID and IC do not form separate clades and are typically assigned based on host (ID is used for eukaryote sequences, while IC is used for prokaryote sequences) (12). Thus, all ID and IC sequences were run through NCBI’s BLAST tool (81) in order to confirm that ID sequences matched known eukaryotic sequences, while IC sequences matched known prokaryotic sequences. If not, they were re-assigned manually.

Pie charts were created based on the coverage of bacterial Rubisco sequences separated by form in all cryopeg brine samples and all sea-ice samples, using the abbreviations from Prywes et al. (12). All form IV subtypes were counted as one form IV category in the charts, as form IV does not participate in autotrophy. Any sequences assigned to forms I or II were also assigned to an organism using BLAST (81).

### MAG assembly

For the reconstruction of MAGs, quality-controlled paired-end Illumina reads of cryopeg brine samples CBIW_0.2_2017, CBIW_3.0_2017, CBIW_2018, CBIA_2018, and CB4_2018 were co-assembled using MEGAHIT v1.2.1 (82). The resulting cryopeg co-assembly was processed with “anvi-script-reformat-fasta” for input into Anvi’o v6.2 (83), retaining only contigs of 2,500 bp and longer. The quality-controlled input reads of each sample were mapped back to the co-assembly using Bowtie2 v2.3.0 (84) to generate sequence alignment map files, which were then converted to a binary format using SAMtools v1.5 (85), and sorted and indexed using “anvi-init-bam” (83).

The co-assembled metagenome contigs were binned using MetaBAT (86), MetaBAT2 v2.15 (87), MaxBin2 v2.2.7 (88), BinSanity v0.4.0 (89), and GroopM2 v0.2.1 (90), and the resulting bin sets were integrated into one combined draft ensemble set using DAS Tool (91), metaWRAP (92), and uniteM (https://github.com/dparks1134/UniteM). The draft ensemble was optimized and de-replicated using another iteration of DAS Tool, metaWRAP, and uniteM to produce three sets of non-redundant draft bins. Bin completion and contamination were determined using CheckM v1.1.3 (93), and only the highest-quality draft bin set was retained. The retained bins were then imported into Anvi’o for visualization and manual curation based on sequence composition and differential coverage (83), and the quality and completion of the refined bins were assessed with CheckM v1.1.3 (93). Only bins of 50%–100% completeness and 0%–10% redundancy (potential contamination) were retained, corresponding to medium-, high-quality, and complete MAGs (94).

### MAG analysis

MAGs were classified taxonomically using the classify_wf pipeline of GTDB-Tk v0.3.2 with database release r89 (95). Prodigal v2.6.3 (96) was used for gene calling as implemented in Anvi’o (83), KofamKOALA v2020-07-01 with KEGG release 95.0 (97) was used to annotate functions with KO, and KEGG decoder v1.2 (98) was used to contextualize KO assignments within metabolic pathways and to assess pathway completion.

Estimates of the relative contribution of MAGs to the total community composition in individual cryopeg brines were calculated based on read mapping to MAGs using CheckM v1.1.3 (93) with the “profile” command. An ANI pairwise comparison using fastANI (99) was carried out to confirm genus identity and determine species identity. ANI values > 95% were considered to indicate a shared species identity.

### *Thiomicrorhabdus* phylogenetics

A total of 45 additional *Thiomicrorhabdus* genomes, including 28 MAGs and 17 isolate genomes, and 2 isolate genomes from a closely related genus, *Hydrogenovibrio*, were obtained from the NCBI database (Table S2). Each genome was analyzed in CheckM (93) using pplacer (100), Prodigal (101), and HMMER v.3.3.2 (http://hmmer.org), for completeness and contamination using as markers the 638 single-copy genes common to the Piscirickettsiaceae family, to which the *Hydrogenovibrio* and *Thiomicrorhabdus* genera both belong. Genomes under 70% completeness or above 5% contamination were discarded. Genomes were also compared pairwise for ANI using fastANI (99). Genomes matching at ≥95% ANI were de-replicated, and the most complete representative was chosen to represent the cluster for phylogenetic analysis. Two genomes that otherwise met these criteria were excluded from further analysis for lack of any complete Rubisco genes.

Functional annotation of the remaining 24 genomes was conducted using the Snakemake (102) “anvi-run-workflow -w contigs” workflow in Anvi’o (83) against the KEGG (103–105), COG (106, 107), and Pfam (108, 109) databases. Environmental data and location of sample were collected from original sources (Table S2). A map of sample locations was created using QGIS 3.30.1 (110) and the ESRI Ocean Basemap (111).

A phylogenetic tree of the *Thiomicrorhabdus* genomes was constructed using the 71 phylogenetically informative marker genes of the Bacteria_71 collection in Anvi’o (83, 112, 113). For each species, nucleotide sequences annotated as these marker genes were extracted from the genome and translated into amino acid sequences using Prodigal (101). In cases where redundant markers were recovered for the same species, the gene with the longest sequence was selected. For each marker, predicted protein sequences were aligned individually using MAFFT (79), and alignments were subsequently trimmed using trimal (-gt 0.1) (114). Next, individual marker alignments were concatenated into a per-species supermatrix. Only those species with 35 or more of the 71 marker genes were retained. Finally, a maximum-likelihood species tree was inferred using IQTree2 with a model selection step and 1,000 ultrafast bootstraps (-bnni -m TEST -st AA -bb 1000) (80). The resulting topology was then visualized in iToL (115) using *Hydrogenovibrio marinus* and *Hydrogenovibrio thermophila* as an outgroup.

### *Thiomicrorhabdus* Rubisco analysis

For each *Thiomicrorhabdus* and *Hydrogenovibrio* genome, all genes annotated as Rubisco large subunits (RbcL) were aligned and trimmed. RbcL genes clustered into three groups, which corresponded to the three known forms (form II, form IAc, and form IAq) from *H. marinus* (62). A manual confirmation of this assignment was performed based on species with known Rubisco forms from Scott et al. (44). Small subunits were identified based on their proximity to large subunits, with an additional manual check to confirm that all form I sequences had a small subunit and a large subunit, and that no form II sequence had a small subunit.

Genome annotations were used to create Rubisco cassette gene maps using ggplot (116) and gggenes (117). A LysR gene next to an RbcL or RbcII gene was considered the beginning of a RbcII or RbcIAq cassette, respectively, while a CynT or CbbO gene was considered the ending of the same. RbcIAc cassettes were considered to start at a RbcLIAc gene and end at a LysR gene. Occasionally, gene rearrangements or contig ends/starts necessitated adjustments to the start or end of the cassette depiction.

### Theoretical model

The carboxylation reaction rate of a Rubisco enzyme (R_C_) is competitively inhibited by O_2_ and can be modeled for each active site under different CO_2_ and O_2_ concentrations using a competitive-inhibitor Michaelis-Menten equation as described in Flamholz et al. (8) (equation 1):


(1) $$R_{C}=k_{cat,C}*\frac{\left[CO_{2}\right]}{\left[CO_{2}\right]+K_{C}+K_{C}*\frac{\left[O_{2}\right]}{K_{O}}}$$

where R_C_ is the gross carboxylation rate of Rubisco, *k*_cat,C_ is the carboxylation rate of each active site, K_C_ and K_O_ are the half saturation constants for CO_2_ and O_2_, respectively, and [CO_2_] and [O_2_] are the concentrations of CO_2_ and O_2_, respectively. This equation can be used to directly compare Rubisco carboxylation of different enzyme forms.

To include the carbon loss due to the PGS pathway, which is the result of the oxygenation reaction, we need to calculate the oxygenation reaction rate (R_O_) using a similar equation (equation 2):


(2) $$R_{O}=k_{cat,O}*\frac{\left[O_{2}\right]}{\left[O_{2}\right]+K_{O}+K_{O}*\frac{\left[CO_{2}\right]}{K_{C}}}$$

The value for *k*_cat,O_ is not measured directly; instead, we utilized the commonly measured specificity factor (S_C/O_), which is the ratio of the carboxylase/oxygenase reaction (equation 3):


(3) $$S_{C/O}=\frac{k_{cat,C}*K_{O}}{k_{cat,O}*K_{C}}$$

The relationship between S_C/O_ and R_C_/R_O_ can be described by equation 4, which can be rearranged to calculate R_O_.


(4) $$\frac{R_{C}}{R_{O}}=S_{C/O}*\frac{\left[CO_{2}\right]}{\left[O_{2}\right]}$$

While Flamholz et al. (8) argue that this relationship only exists during low CO_2_ and O_2_ conditions, when CO_2_ << K_C_ and O_2_ << K_O_, we find, in agreement with the original derivation by Laing et al. (118), that this relationship is true for any CO_2_ or O_2_ concentration (see supplemental Methods for full derivation).

To include the loss of carbon to PGS in the model, *Thiomicrorhabdus* genomes were searched for genes matching the key genes of the four known PGS pathways (52). No *Thiomicrorhabdus* genome contained a gene annotated as malate synthase [EC 2.3.3.9], CoA-acylating glyoxylate decarboxylase [EC 1.2.1.17], or glyoxylate carboligase [EC 4.1.1.47], the key genes of the malate cycle, oxalyl-CoA decarboxylation route, and the glycerate pathway, respectively. Some key enzymes of the C2 cycle were present in most *Thiomicrorhabdus* genomes, including serine hydroxymethyltransferase [EC 2.1.2.45] (18/25) and the glycine cleavage system L [EC 1.8.1.4] (24/25) and H proteins (23/25). Therefore, *Thiomicrorhabdus* was assumed to use the C2 cycle for modeling the stoichiometry of PGS carbon loss.

The C2 cycle results in a loss of one CO_2_ molecule for every two 2-PG molecules fixed (52). Using this stoichiometry, the net carboxylation rate (*f*) is:


(5) $$\begin{array}{c} f_{1}=R_{C}-0.5*R_{O}\\ f_{1}=R_{C}-0.5*\frac{R_{C}}{S_{C/O}}*\frac{\left[O_{2}\right]}{\left[{CO}_{2}\right]} \end{array}$$

A model for the gross Rubisco carboxylation rate and the net Rubisco carboxylation including the PGS pathway was created for each Rubisco. A rate of 0 or less denoted that autotrophy could not occur under the specified conditions.

To include temperature effects in the model as well as the effect of CO_2_ and O_2_ concentrations, an Arrhenius-type equation with the form $x\left(T\right)=e^{c-\frac{\Delta H}{RT}}$ was created for each kinetic parameter (*k*_cat,C_, K_C_, K_O_, and *k*_cat,O_) for each enzyme, as in Galmés et al. (26). For each equation, an appropriate ΔH was taken from reported values in Galmés et al. (26), while the constant *c* was calculated by setting the equation equal to a reported value for each kinetic parameter. *R* is the universal gas constant, 0.008314 kJ/mol/K.

The following shows an example calculation for the *k*_cat,C_ for form IAc:

The reported value for the *H. marinus* form IAc *k*_cat,C_ is 2.98 at 30°C (51), and the ΔH chosen for form IA *k*_cat,C_ is 47.2 kJ/mol (an average of three proteobacteria values) (11). Thus:


$$\begin{array}{c} 2.98=e^{c-\frac{47.2}{0.008314*303.15}}\\ c=\ln(2.98)+18.73=19.83\\ k_{cat,C}(T)=e^{19.83-\frac{47.2}{RT}} \end{array}$$

Values used for this analysis are available in Tables S3 and S4, but briefly: reported kinetic parameters were taken from Flamholz et al. (8), Davidi et al. (50), and Hayashi et al. (51) (Table S5); ΔH values were taken from Galmés et al. (11, 26) (Table S4). As the studies that reported ΔH values for form IA enzymes did not distinguish between form IAc and form IAq enzymes, the form IAc and form IAq models used the same ΔH values.

To compare Rubisco forms, the rate of the form IAc Rubisco was subtracted from form II. When the resulting equation has a positive value, form II is faster than form IAc, and vice versa.

### Rubisco thermal adaptation

There are a number of amino acid substitutions that are associated with either cold or hot adaptation (119), and while these may suggest possible thermal adaptation, they do not guarantee it (53). Thus, amino acid sequences of Rubisco were interrogated for signals of potential thermal adaptation using the methods from Raymond-Bouchard et al. (53). Ten indices were incorporated to calculate an adaptive score: number of glycines, number of prolines, number of serines, number of acidic residues (D,E), number of charged residues (D,E,H,K,R), number of polar uncharged residues (C,N,Q,S,T,Y), aliphatic index, aromaticity, and hydrophobicity. Rubisco sequences included the large and small subunits, where relevant, and counts of all amino acids, as well as aromaticity, hydrophobicity (using Grand Average of Hydropathicity index, hereafter GRAVY), and isoelectric point were calculated using the Biopython (120) module Prot.Param, modeled on the Expasy ProtParam tool (121) for each sequence. Aliphatic index was calculated as in Ikai (122).

A reference (mesophilic) value for each index, within each Rubisco form (IAq, IAc, and II), was determined as the average and standard error value of Rubiscos from five genomes (*H. marinus, Thiomicrorhabdus cannonii, Thiomicrorhabdus marina* (previously 6S2-11)*, Thiomicrorhabdus heinhorstiae,* and *T. sediminis*) based on their known mesophilic temperature ranges. Only three Rubisco sequences were used to generate the reference values for form IAq, as *T. sediminis* and *T*. *lithotrophica* do not contain form IAq Rubisco. A one-sample *t*-test was used to determine if the test sequence index value was significantly different from the mesophilic reference values. One form IAq index had a standard error of 0 for the reference values; as a *P* value could not be calculated, any change was considered to be significant. Cold or hot adaptation was assigned to significant indices based on direction of change (Table S10).

An index with a significant value in the cold direction scored as +1, and in the hot direction as –1. Those with no significance scored 0. The total score of all 10 indices was summed to receive an overall thermal adaptation score. Values between –2 and 2 were considered neutral. Values ≥ 3 indicated cold adaptation, while values ≤ –3 indicated hot adaptation.

### Predicted Rubisco structure

Sequences of *H. marinus* form II Rubisco, three neutral form II Rubiscos (from *T. frisia* Kp2, *T. indica*, and *T. aquaedulcis*) and three cold-inferred form II Rubiscos (*T. arctica* UC1, *T*. sp. NP51, and *T. chilensis*) were used to build hypothetical Rubisco dimer protein structures using the AlphaFold (54, 55) multimer model. Resulting Protein Data Bank files were visualized in ChimeraX (123) and were aligned in space using the in-built alignment tool (only paired atoms). Amino acids associated with the active sites were highlighted in red for each protein. Amino acids in *H. marinus* missing in UC1 and all other “cold” Rubisco form II were highlighted in yellow. In addition, amino acid changes in UC1 where the function of the amino acid changed significantly, i.e., where the BLOSUM62 matrix identifies the change as being semi-conservative or not conservative (consensus symbol “.” or empty on the CLUSTAL description), were highlighted in orange.

## Data Availability

Metagenomic data used for the identification and extraction of Rubisco sequences from cryopeg brines and sea-ice samples are available under SRA BioProject accession numbers PRJNA570544, PRJNA539724, PRJNA539309, PRJNA539308, PRJNA539600, PRJNA539601, PRJNA518292, PRJNA518293, PRJNA570545, PRJNA518295, and PRJNA518294. Cryopeg brine metagenomes used for the generation of MAGs are available at PRJNA539724, PRJNA539309, PRJNA539308, PRJNA539600, and PRJNA539601. Autotrophic cryopeg brine MAGs are available for download at https://doi.org/10.6084/m9.figshare.28972226. All other publicly available genome sequences used for this study can be found in NCBI under the accession numbers listed in Table S1.

## SUPPLEMENTAL MATERIAL

Supplemental material aem.00604-25-s0001.docx Supplemental methods, Figures S1 to S10, and Tables S1 to S10.

ASM does not own the copyrights to Supplemental Material that may be linked to, or accessed through, an article. The authors have granted ASM a non-exclusive, world-wide license to publish the Supplemental Material files. Please contact the corresponding author directly for reuse.
