# Supplementary material for: Chemoautotrophy in subzero environments and the potential for cold-adapted Rubisco
Source: Appl Environ Microbiol. 2025 May 30;91(6):e00604-25. doi: 10.1128/aem.00604-25 (PMC12175532; doi:10.1128/aem.00604-25)
Supplement: Supplemental material — Supplemental methods, Figures S1 to S10, and Tables S1 to S10. [file aem.00604-25-s0001.docx]

**Supplemental Texts:**

| Supplemental Methods | 1 |
| --- | --- |
| Supplemental Figures 1-10 | 2 |
| Supplemental Tables 1-10 | 12 |
| Supplemental References | 23 |

**Supplemental Methods:**

The derivation of Equation 4 from Laing et al. 1974 (1) is reproduced here:

$$\frac{R_{C}}{R_{O}}=\frac{k_{cat,C}*\frac{\left[ CO_{2} \right]}{\left[ CO_{2} \right]+K_{C}+K_{C}*\frac{\left[ O_{2} \right]}{K_{O}}}}{k_{cat,O}*\frac{\left[ O_{2} \right]}{\left[ O_{2} \right]+K_{O}+K_{O}*\frac{\left[ CO_{2} \right]}{K_{C}}}}$$

$$\frac{R_{C}}{R_{O}}=\frac{k_{cat,C}}{k_{cat,O}}*\frac{\left[ CO_{2} \right]}{\left[ O_{2} \right]}*\frac{\left[ O_{2} \right]+K_{O}+K_{O}*\frac{\left[ CO_{2} \right]}{K_{C}}}{\left[ CO_{2} \right]+K_{C}+K_{C}*\frac{\left[ O_{2} \right]}{K_{O}}}$$

$$\frac{R_{C}}{R_{O}}=\frac{k_{cat,C}}{k_{cat,O}}*\frac{\left[ CO_{2} \right]}{\left[ O_{2} \right]}*\frac{\left[ O_{2} \right]*\frac{K_{C}}{K_{C}}+K_{O}*\frac{K_{C}}{K_{C}}+K_{O}*\frac{\left[ CO_{2} \right]}{K_{C}}}{\left[ CO_{2} \right]*\frac{K_{O}}{K_{O}}+K_{C}*\frac{K_{O}}{K_{O}}+K_{C}*\frac{\left[ O_{2} \right]}{K_{O}}}$$

$$\frac{R_{C}}{R_{O}}=\frac{k_{cat,C}}{k_{cat,O}}*\frac{\left[ CO_{2} \right]}{\left[ O_{2} \right]}*\frac{\frac{\left[ O_{2} \right]*K_{C}+K_{O}*K_{C}+K_{O}*\left[ CO_{2} \right]}{K_{C}}}{\frac{\left[ CO_{2} \right]*K_{O}+K_{C}*K_{O}+K_{C}*\left[ O_{2} \right]}{K_{O}}}$$

$$\frac{R_{C}}{R_{O}}=\frac{k_{cat,C}}{k_{cat,O}}*\frac{\left[ CO_{2} \right]}{\left[ O_{2} \right]}*\frac{K_{O}}{K_{C}}*\frac{\left[ O_{2} \right]*K_{C}+K_{O}*K_{C}+K_{O}*\left[ CO_{2} \right]}{K_{C}*\left[ O_{2} \right]+K_{C}*K_{O}+\left[ CO_{2} \right]*K_{O}}$$

$$\frac{R_{C}}{R_{O}}=\frac{k_{cat,C}}{k_{cat,O}}*\frac{\left[ CO_{2} \right]}{\left[ O_{2} \right]}*\frac{K_{O}}{K_{C}}=\frac{k_{cat,C}*K_{O}}{k_{cat,O}*K_{C}}*\frac{\left[ CO_{2} \right]}{\left[ O_{2} \right]}=S_{C/O}*\frac{\left[ CO_{2} \right]}{\left[ O_{2} \right]}$$

**Supplemental Figures:**


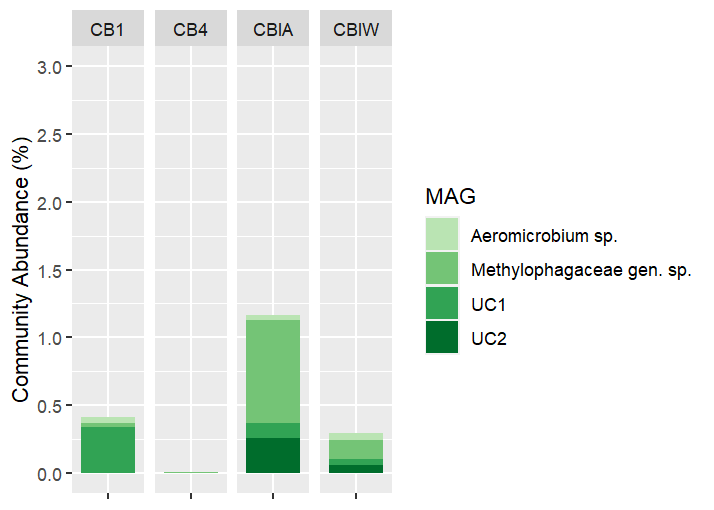


Fig. S1: Autotrophic community member abundance in the cryopeg brine metagenomes.

Abundance calculated as percentage of total reads mapped to the MAG.

| **T. arctica UC1** | 100 | 86 |  |  |  |  |  |  | 99 |  |  |  |  |  |  |  |  |  |  |  |  |  |  | 84 |  |  |  |  |  |  |  |  |  |  |  |  |  |  |  |  |  |  |  |  |  | 85 |  |
| --- | --- | --- | --- | --- | --- | --- | --- | --- | --- | --- | --- | --- | --- | --- | --- | --- | --- | --- | --- | --- | --- | --- | --- | --- | --- | --- | --- | --- | --- | --- | --- | --- | --- | --- | --- | --- | --- | --- | --- | --- | --- | --- | --- | --- | --- | --- | --- |
| **T. sp. UC2** | 86 | 100 |  |  |  |  |  |  | 86 |  |  |  |  |  |  |  |  |  |  |  |  |  |  | 90 |  |  |  |  |  |  |  |  |  |  |  |  |  |  |  |  |  |  |  |  |  | 90 |  |
| **T. sp. 6S2-11** |  |  | 100 |  |  |  |  |  |  |  |  |  |  |  |  |  |  |  |  |  |  |  |  |  |  |  |  |  |  |  |  |  |  |  |  |  |  |  |  |  |  |  |  |  |  |  |  |
| **T. sp. 6S3-12** |  |  |  | 100 |  |  |  |  |  |  |  |  |  |  |  |  |  |  |  |  |  |  |  |  |  |  |  |  |  |  |  |  |  |  |  |  |  |  |  |  |  | 92 |  |  |  |  |  |
| **T. immobilis (Am19)** |  |  |  |  | 100 | 80 | 80 |  |  |  |  |  |  |  |  |  |  |  |  |  |  |  |  |  |  |  |  |  |  |  |  |  |  |  |  |  |  |  |  |  |  |  | 80 |  |  |  |  |
| **T. frisia Kp2** |  |  |  |  | 80 | 100 |  |  |  |  |  |  |  |  |  |  |  |  |  |  |  |  |  |  |  |  |  |  |  |  |  |  |  |  |  |  |  |  |  |  |  |  |  |  |  |  |  |
| **T. sp. Milos-T2** |  |  |  |  | 80 |  | 100 |  |  |  |  |  |  |  |  |  |  |  |  |  |  |  |  |  |  |  |  |  |  |  |  |  |  |  |  |  |  |  |  |  |  |  | 80 |  |  |  |  |
| **T. aquaedulcis** |  |  |  |  |  |  |  | 100 |  |  |  |  |  |  |  |  |  |  |  |  |  |  |  |  |  |  |  |  |  |  |  |  |  |  |  |  |  |  |  |  |  |  |  |  |  |  |  |
| **T. arctica** | 99 | 86 |  |  |  |  |  |  | 100 |  |  |  |  |  |  |  |  |  |  |  |  |  |  | 85 |  |  |  |  |  |  |  |  |  |  |  |  |  |  |  |  |  |  |  |  |  | 85 |  |
| **T. cannonii** |  |  |  |  |  |  |  |  |  | 100 |  |  |  |  |  |  |  |  |  |  |  |  |  |  |  |  |  |  |  |  |  |  |  |  |  |  |  |  |  |  |  |  |  |  |  |  |  |
| **T. chilensis** |  |  |  |  |  |  |  |  |  |  | 100 |  |  |  |  |  |  |  |  |  |  |  |  |  |  |  |  |  |  |  |  |  |  |  |  |  |  |  |  |  |  |  |  |  |  |  |  |
| **T. heinhorstiae** |  |  |  |  |  |  |  |  |  |  |  | 100 |  |  |  |  |  |  |  |  |  |  |  |  |  |  |  |  |  |  |  |  |  |  |  |  |  |  |  |  |  |  |  |  |  |  |  |
| **T. indica** |  |  |  |  |  |  |  |  |  |  |  |  | 100 |  |  |  |  |  |  |  |  |  |  |  |  |  |  |  |  |  |  |  |  |  |  |  |  |  |  |  |  |  |  |  |  |  |  |
| **T. sediminis** |  |  |  |  |  |  |  |  |  |  |  |  |  | 100 |  |  |  |  |  |  |  |  |  |  |  |  |  |  |  |  |  |  |  |  |  |  |  |  |  |  |  |  |  |  |  |  |  |
| T. sp. 354-166_108 |  |  |  |  |  |  |  |  |  |  |  |  |  |  | 100 | 99 |  |  | 81 | 81 |  |  |  |  | 81 |  |  | 81 |  | 81 |  |  |  |  |  | 81 |  |  | 80 |  |  |  |  |  |  |  |  |
| T. sp. 355-202_026 |  |  |  |  |  |  |  |  |  |  |  |  |  |  | 99 | 100 |  |  | 81 | 81 |  |  |  |  | 81 |  |  | 81 |  | 80 |  |  |  |  |  | 81 |  |  | 81 |  |  |  |  |  |  |  |  |
| T. sp. 4281-140_065 |  |  |  |  |  |  |  |  |  |  |  |  |  |  |  |  | 100 |  | 80 |  | 85 |  | 82 |  | 80 |  | 82 |  |  |  | 82 | 82 |  | 81 | 82 |  |  |  |  | 82 | 81 |  |  |  |  |  |  |
| T. sp. A3_174 |  |  |  |  |  |  |  |  |  |  |  |  |  |  |  |  |  | 100 |  |  |  | 99 | 81 |  | 82 | 98 |  | 82 | 97 | 82 |  | 80 | 96 |  |  | 82 | 97 | 98 | 80 |  |  |  |  |  |  |  |  |
| T. sp. D100SM_12 |  |  |  |  |  |  |  |  |  |  |  |  |  |  | 81 | 81 | 80 |  | 100 | 96 | 85 |  |  |  | 81 |  |  | 81 |  | 80 |  |  |  |  | 80 | 80 |  |  | 80 |  |  |  |  |  |  |  |  |
| T. sp. D96SM_37 |  |  |  |  |  |  |  |  |  |  |  |  |  |  | 81 | 81 |  |  | 96 | 100 | 81 |  |  |  | 81 |  |  | 81 |  |  |  |  |  |  |  | 80 |  |  | 80 |  |  |  |  |  |  |  |  |
| **T. sp. D96SM_15** |  |  |  |  |  |  |  |  |  |  |  |  |  |  |  |  | 85 |  | 85 | 81 | 100 |  | 81 |  |  |  | 81 |  |  |  | 81 | 81 |  | 81 | 81 |  |  |  |  | 81 |  |  |  |  |  |  |  |
| T. sp. M17_146 |  |  |  |  |  |  |  |  |  |  |  |  |  |  |  |  |  | 99 |  |  |  | 100 |  |  | 82 | 98 |  | 82 | 97 | 82 |  | 80 | 97 |  |  | 82 | 97 | 98 |  | 81 |  |  |  |  |  |  |  |
| T. sp. M17_171 |  |  |  |  |  |  |  |  |  |  |  |  |  |  |  |  | 82 | 81 |  |  | 81 |  | 100 |  | 82 | 80 | 98 | 82 |  | 81 | 98 | 98 |  | 98 | 98 | 81 |  | 80 | 81 | 98 | 87 |  |  |  |  |  |  |
| **T. sp. NP51** | 84 | 90 |  |  |  |  |  |  | 85 |  |  |  |  |  |  |  |  |  |  |  |  |  |  | 100 |  |  |  |  |  |  |  |  |  |  |  |  |  |  |  |  |  |  |  |  |  | 99 |  |
| T. sp. S012_012 |  |  |  |  |  |  |  |  |  |  |  |  |  |  | 81 | 81 | 80 | 82 | 81 | 81 |  | 82 | 82 |  | 100 | 82 |  | 99 | 82 | 95 | 81 | 83 | 82 | 81 | 82 | 97 | 81 | 82 | 98 | 82 |  |  |  |  |  |  |  |
| T. sp. S012_085 |  |  |  |  |  |  |  |  |  |  |  |  |  |  |  |  |  | 98 |  |  |  | 98 | 80 |  | 82 | 100 |  | 82 |  | 82 |  | 80 | 99 |  |  | 83 | 99 | 100 | 80 |  |  |  |  |  |  |  |  |
| T. sp. S012_107 |  |  |  |  |  |  |  |  |  |  |  |  |  |  |  |  | 82 |  |  |  | 81 |  | 98 |  |  |  | 100 |  |  |  | 98 | 100 |  | 97 | 99 |  |  |  |  | 99 | 86 |  |  |  |  |  |  |
| **T. sp. S012_112** |  |  |  |  |  |  |  |  |  |  |  |  |  |  | 81 | 81 |  | 82 | 81 | 81 |  | 82 | 82 |  | 99 | 82 |  | 100 | 82 | 95 | 81 | 83 | 82 | 81 | 81 | 81 | 97 | 83 | 98 | 82 |  |  |  |  |  |  |  |
| T. sp. S012_68 |  |  |  |  |  |  |  |  |  |  |  |  |  |  |  |  |  | 97 |  |  |  | 97 |  |  | 82 |  |  | 82 | 100 | 83 |  | 80 | 99 |  |  | 82 | 99 | 100 | 81 |  |  |  |  |  |  |  |  |
| T. sp. S013_057_VB |  |  |  |  |  |  |  |  |  |  |  |  |  |  | 81 | 80 |  | 82 | 80 |  |  | 82 | 81 |  | 95 | 82 |  | 95 | 83 | 100 | 81 | 83 | 82 | 81 | 82 | 94 | 82 | 83 | 94 | 81 |  |  |  |  |  |  |  |
| T. sp. S013_18 |  |  |  |  |  |  |  |  |  |  |  |  |  |  |  |  | 82 |  |  |  | 81 |  | 98 |  | 81 |  | 98 | 81 |  | 81 | 100 | 100 |  | 97 | 99 | 81 |  |  | 80 | 99 | 86 |  |  |  |  |  |  |
| T. sp. S013_67 |  |  |  |  |  |  |  |  |  |  |  |  |  |  |  |  | 82 | 80 |  |  | 81 | 80 | 98 |  | 83 | 80 | 100 | 83 | 80 | 83 | 100 | 100 |  | 99 | 99 | 82 |  | 80 | 82 | 99 | 87 |  |  |  |  |  |  |
| T. sp. S139_146 |  |  |  |  |  |  |  |  |  |  |  |  |  |  |  |  |  | 96 |  |  |  | 97 |  |  | 82 | 99 |  | 82 | 99 | 82 |  |  | 100 |  |  | 82 | 99 | 100 | 80 |  |  |  |  |  |  |  |  |
| T. sp. S139_169 |  |  |  |  |  |  |  |  |  |  |  |  |  |  |  |  | 81 |  |  |  | 81 |  | 98 |  | 81 |  | 97 | 81 |  | 81 | 97 | 99 |  | 100 | 98 | 81 |  |  | 81 | 99 | 86 |  |  |  |  |  |  |
| T. sp. S140_061 |  |  |  |  |  |  |  |  |  |  |  |  |  |  |  |  | 82 |  | 80 |  | 81 |  | 98 |  | 82 |  | 99 | 81 |  | 82 | 99 | 99 |  | 98 | 100 | 80 |  | 80 | 81 | 99 | 87 |  |  |  |  |  |  |
| T. sp. S140_168 |  |  |  |  |  |  |  |  |  |  |  |  |  |  | 81 | 81 |  | 82 | 80 | 80 |  | 82 | 81 |  | 97 | 83 |  | 81 | 82 | 94 | 81 | 82 | 82 | 81 | 80 | 100 | 81 | 84 | 97 | 80 |  |  |  |  |  |  |  |
| T. sp. S140_198 |  |  |  |  |  |  |  |  |  |  |  |  |  |  |  |  |  | 97 |  |  |  | 97 |  |  | 81 | 99 |  | 97 | 99 | 82 |  |  | 99 |  |  | 81 | 100 | 100 | 80 |  |  |  |  |  |  |  |  |
| **T. sp. S141_101** |  |  |  |  |  |  |  |  |  |  |  |  |  |  |  |  |  | 98 |  |  |  | 98 | 80 |  | 82 | 100 |  | 83 | 100 | 83 |  | 80 | 100 |  | 80 | 84 | 100 | 100 | 81 |  |  |  |  |  |  |  |  |
| T. sp. S141_143 |  |  |  |  |  |  |  |  |  |  |  |  |  |  | 80 | 81 |  | 80 | 80 | 80 |  |  | 81 |  | 98 | 80 |  | 98 | 81 | 94 | 80 | 82 | 80 | 81 | 81 | 97 | 80 | 81 | 100 | 81 |  |  |  |  |  |  |  |
| **T. sp. S141_227** |  |  |  |  |  |  |  |  |  |  |  |  |  |  |  |  | 82 |  |  |  | 81 | 81 | 98 |  | 82 |  | 99 | 82 |  | 81 | 99 | 99 |  | 99 | 99 | 80 |  |  | 81 | 100 | 87 |  |  |  |  |  |  |
| T. sp. UWMA-0242 |  |  |  |  |  |  |  |  |  |  |  |  |  |  |  |  | 81 |  |  |  |  |  | 87 |  |  |  | 86 |  |  |  | 86 | 87 |  | 86 | 87 |  |  |  |  | 87 | 100 |  |  |  |  |  |  |
| **T. xiamenesis** |  |  |  | 92 |  |  |  |  |  |  |  |  |  |  |  |  |  |  |  |  |  |  |  |  |  |  |  |  |  |  |  |  |  |  |  |  |  |  |  |  |  | 100 |  |  |  |  |  |
| **T. sp. XGS_01** |  |  |  |  | 80 |  | 80 |  |  |  |  |  |  |  |  |  |  |  |  |  |  |  |  |  |  |  |  |  |  |  |  |  |  |  |  |  |  |  |  |  |  |  | 100 |  |  |  |  |
| **T. sp. zzn3** |  |  |  |  |  |  |  |  |  |  |  |  |  |  |  |  |  |  |  |  |  |  |  |  |  |  |  |  |  |  |  |  |  |  |  |  |  |  |  |  |  |  |  | 100 |  |  |  |
| **T. sp. ZW0627** |  |  |  |  |  |  |  |  |  |  |  |  |  |  |  |  |  |  |  |  |  |  |  |  |  |  |  |  |  |  |  |  |  |  |  |  |  |  |  |  |  |  |  |  | 100 |  |  |
| **T. sp. GH6** | 85 | 90 |  |  |  |  |  |  | 85 |  |  |  |  |  |  |  |  |  |  |  |  |  |  | 99 |  |  |  |  |  |  |  |  |  |  |  |  |  |  |  |  |  |  |  |  |  | 100 |  |
| **T. zosterae** |  |  |  |  |  |  |  |  |  |  |  |  |  |  |  |  |  |  |  |  |  |  |  |  |  |  |  |  |  |  |  |  |  |  |  |  |  |  |  |  |  |  |  |  |  |  | 100 |
|  | **T. arctica UC1** | **T. sp. UC2** | **T. sp. 6S2-11** | **T. sp. 6S3-12** | **T. immobilis (Am19)** | **T. frisia Kp2** | **T. sp. Milos_T2** | **T. aquaedulcis** | **T. arctica** | **T. cannonii** | **T. chilensis** | **T. heinhorstiae** | **T. indica** | **T. sediminis** | T. sp. 354-166_108 | T. sp. 355-202_026 | T. sp. 4281-140_065 | T. sp. A3_174 | T. sp. D100SM_12 | T. sp. D96SM_37 | **T. sp. D96SM_15** | T. sp. M17_146 | T. sp. M17_171 | **T. sp. NP51** | T. sp. S012_012 | T. sp. S012_085 | T. sp. S012_107 | **T. sp. S012_112** | T. sp. S012_68 | T. sp. S013_057_VB | T. sp. S013_18 | T. sp. S013_67 | T. sp. S139_146 | T. sp. S139_169 | T. sp. S140_061 | T. sp. S140_168 | T. sp. S140_198 | **T. sp. S141_101** | T. sp. S141_143 | **T. sp. S141_227** | T. sp. UWMA-0242 | **T. xiamenesis** | **T. sp. XGS-01** | **T. sp. zzn3** | **T. sp. ZW0627** | **T. sp. GH6** | **T. zosterae** |

Fig. S2: ANI comparison of all known *Thiomicrorhabdus* genomes.

<80% ANI not pictured, as ANI becomes unreliable at less than 80% identity. Boxes shaded green indicate that the two genomes had >95% ANI, so represent the same species. Bolded genomes were used in further analysis.


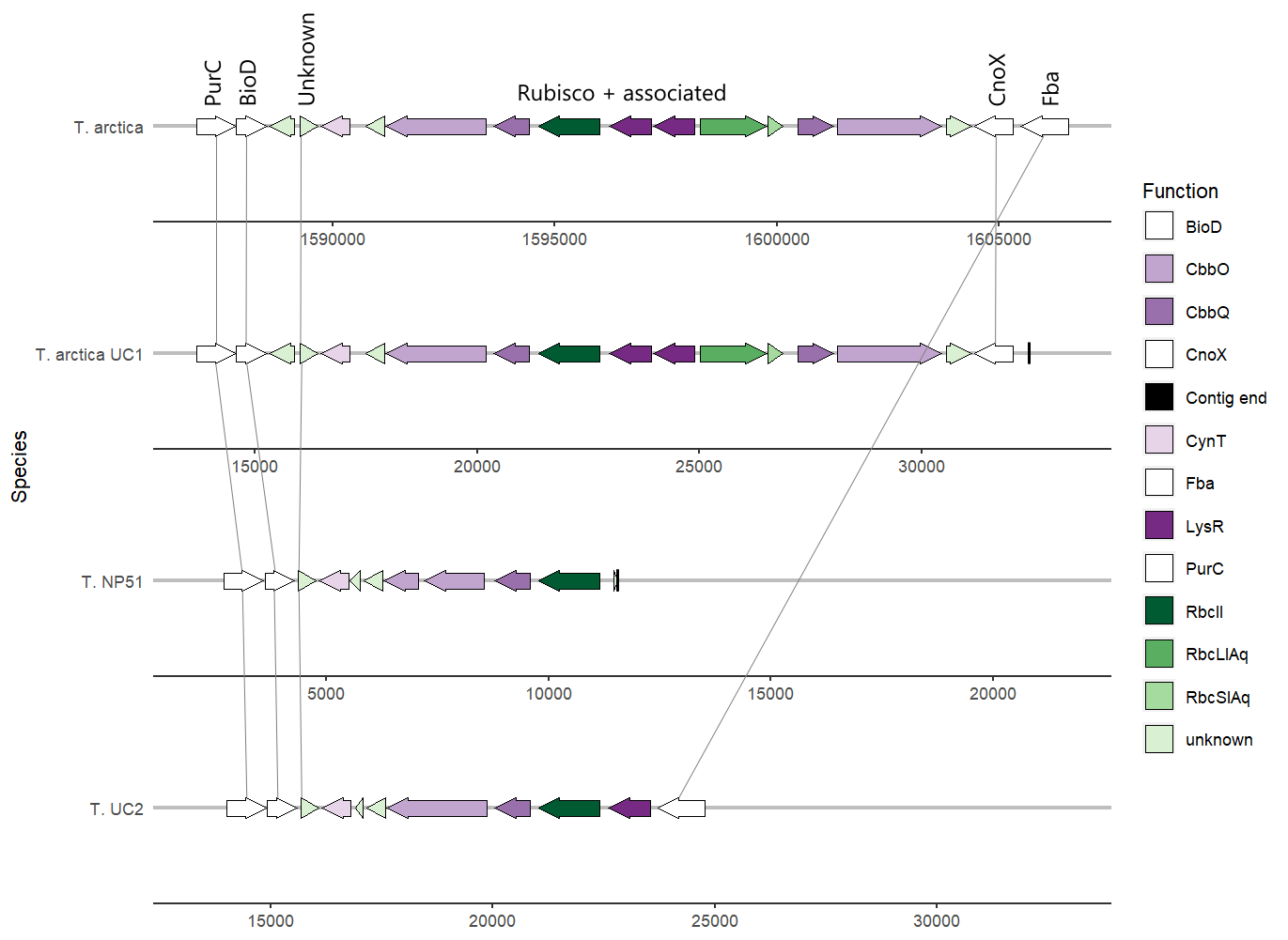


Fig. S3: Gene maps of Rubisco cassette in cryopeg brine MAGs and relatives.

Numbers along bottom of scale represent bp in genome or contig. Genes colored according to function: green=Rubisco genes, purple=Rubisco-associated genes, light green = unknown function, white = other genes).


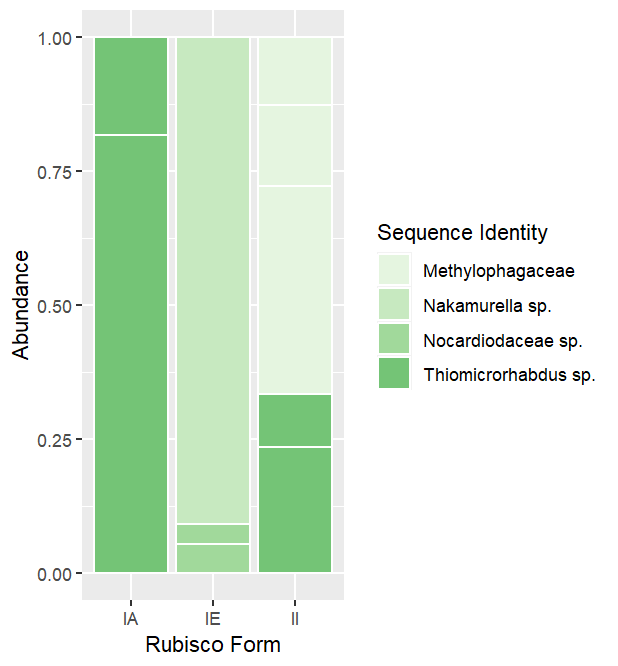

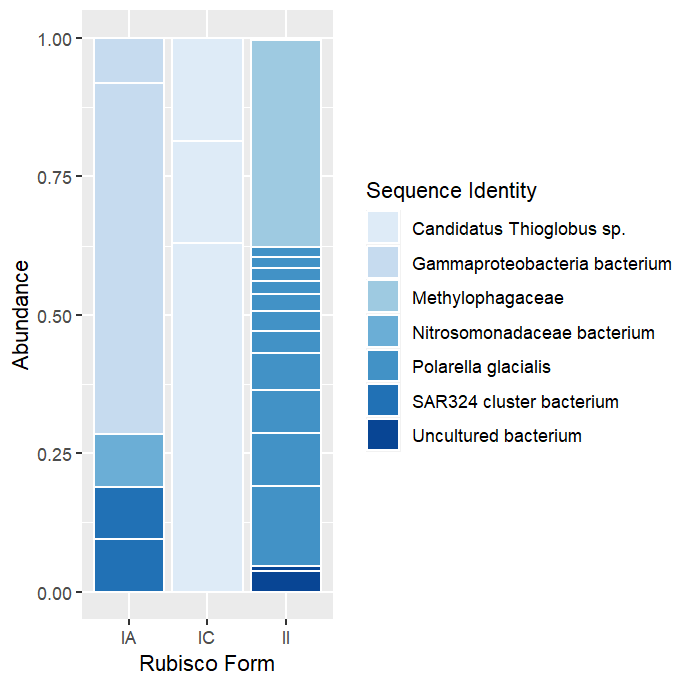


Fig. S4: Distinct sequence identity and abundance in cryopeg brine (left) and sea-ice (right) samples

Read depth of each distinct sequence (ones that differed in at least one amino acid) per Rubisco form. Organism identity assigned using the NCBI BLAST algorithm.


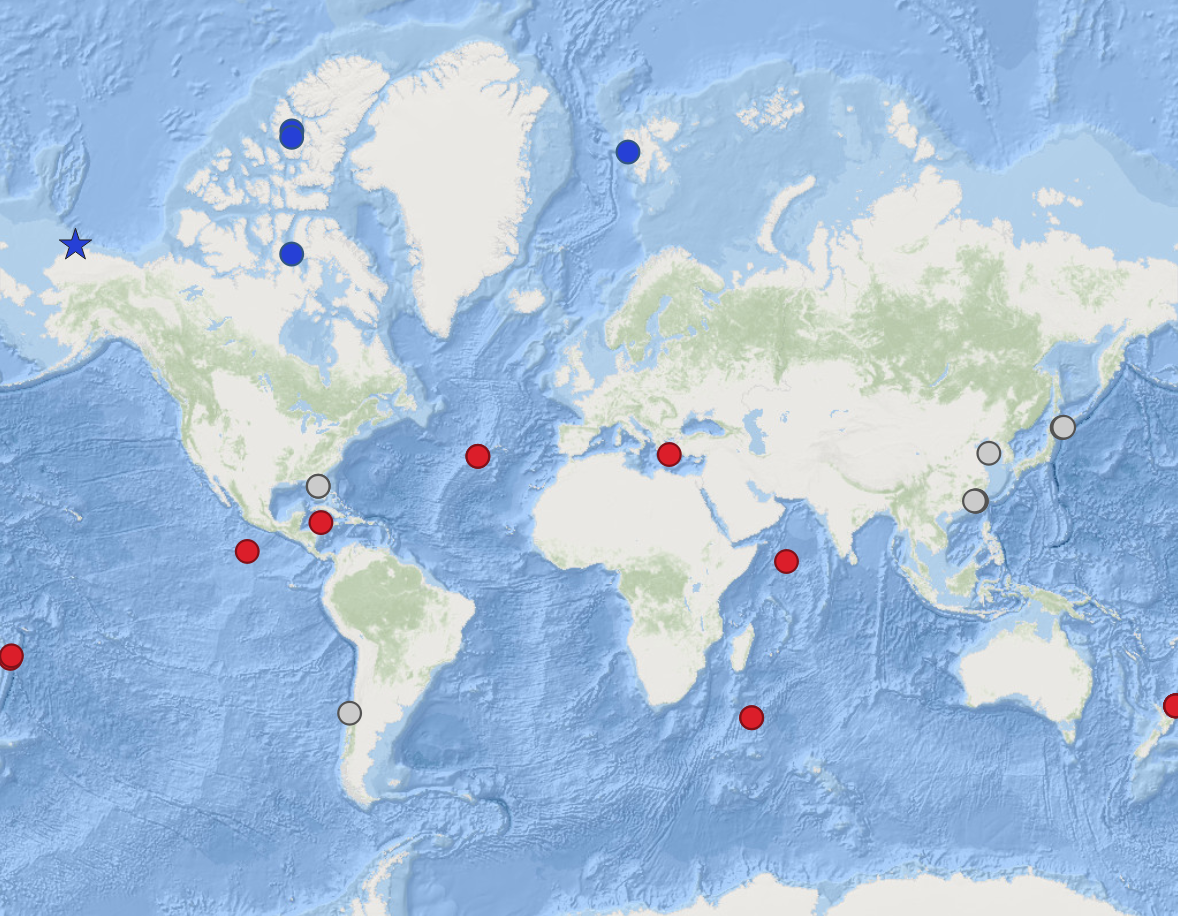


Fig. S5: Map of *Thiomicrorhabdus* genome sampling locations.

Colors denote habitat type: red for hydrothermal vents, blue for polar, and grey for other. The blue star indicates the location of the cryopeg brines analyzed in this study.


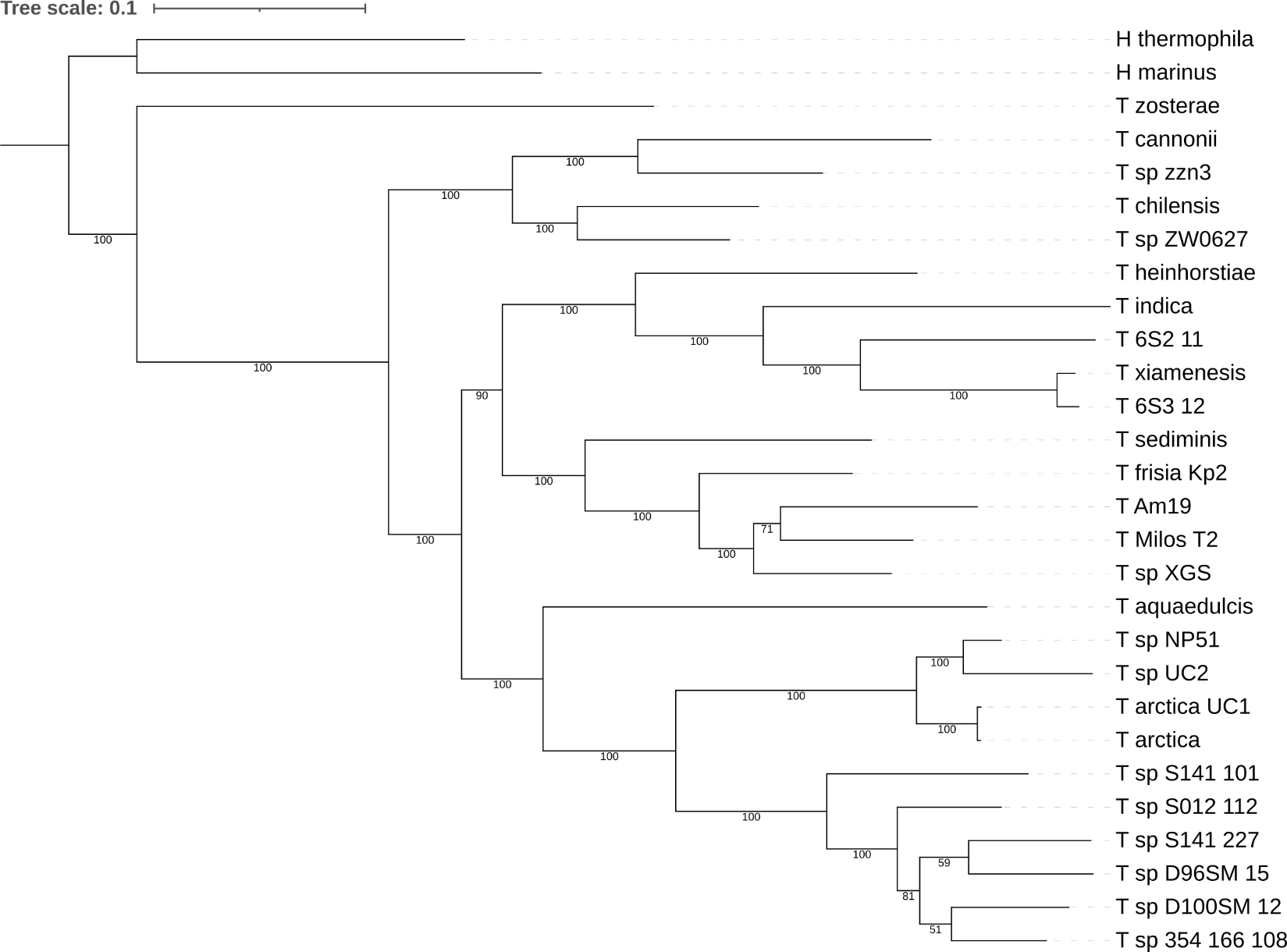


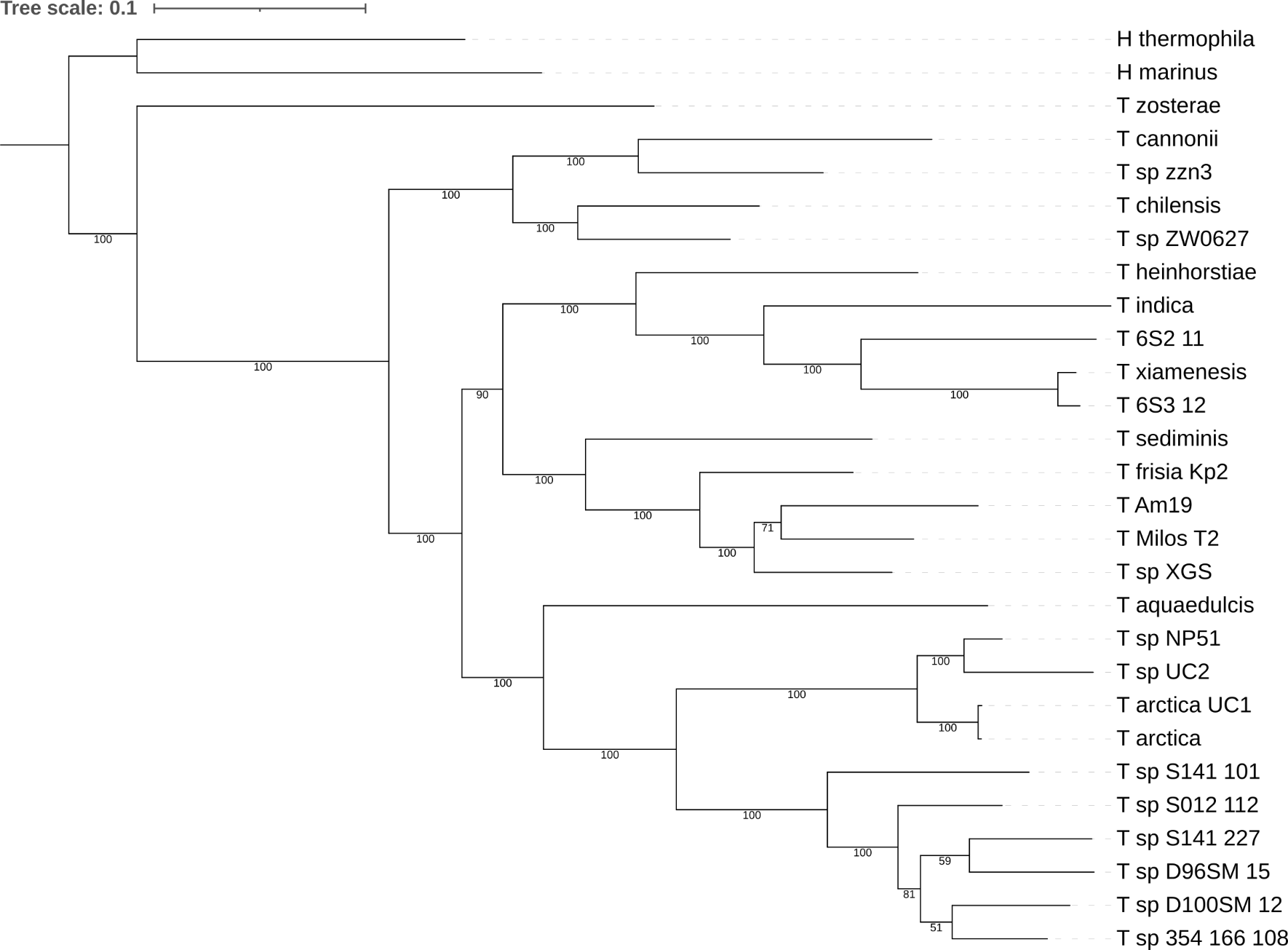


Fig. S6: Expanded Clade 3 from *Thiomicrorhabdus* phylogenetic tree.

Section of *Thiomicrorhabdus* phylogenetic tree including two MAGs that passed quality control but did not contain a full Rubisco sequence. Branch leads to the rest of the tree as visualized in Fig. 2.


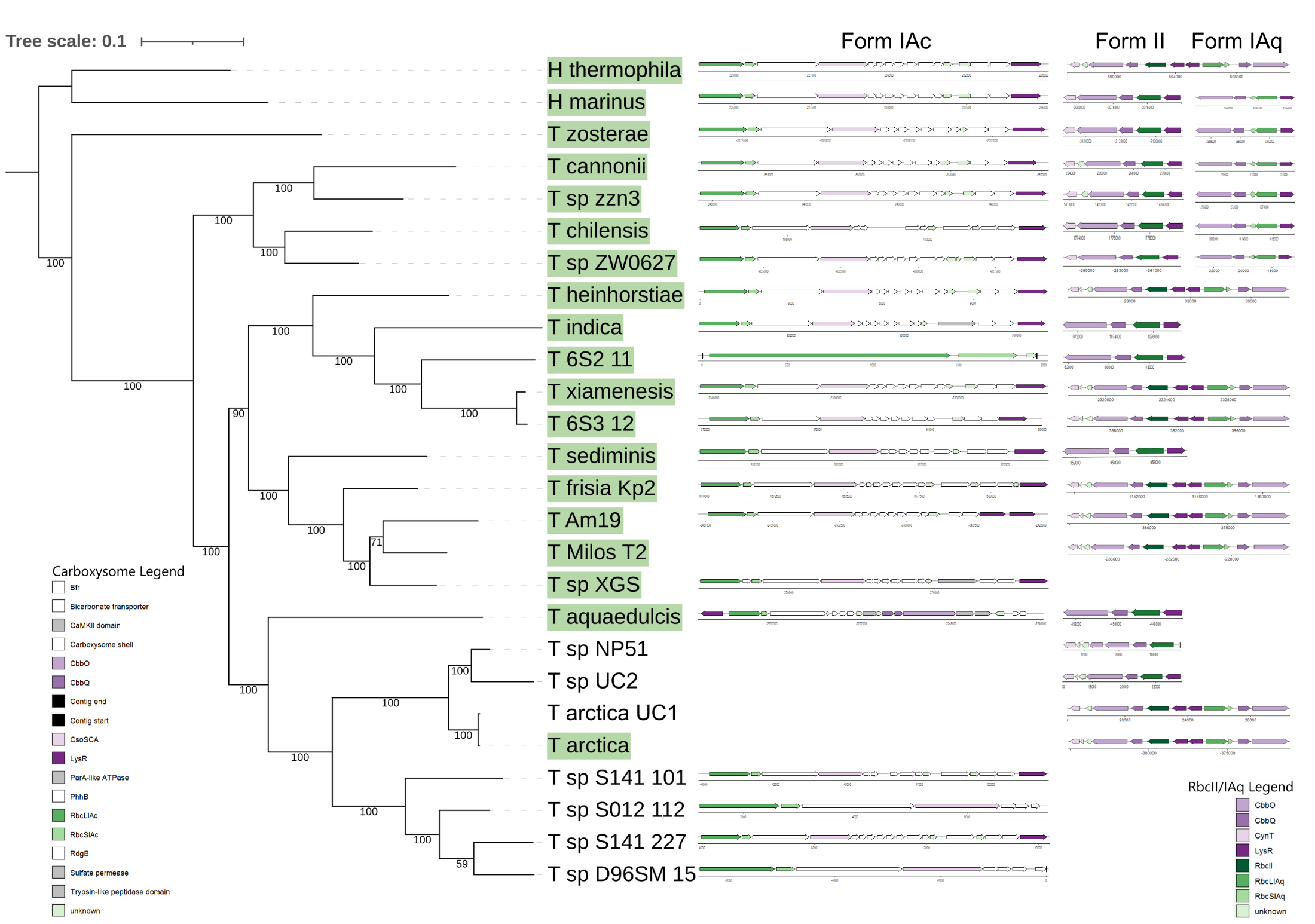


**B**

**A**

Fig. S7: Gene maps of Rubisco cassettes.

Arrangement of genes around the Rubisco cassettes of *Thiomicrorhabdus* genomes. Phylogenetic tree reproduced from Fig. 3. A) Form IAc (carboxysome) cassettes (legend on left), and B) form II/form IAq cassettes (legend on right). Numbers along the bottom of scale depict bp in genome. Genes are colored based on function: green = Rubisco genes, purple = Rubisco-associated genes, white = carboxysome genes, grey = other genes, light green = unknown function.


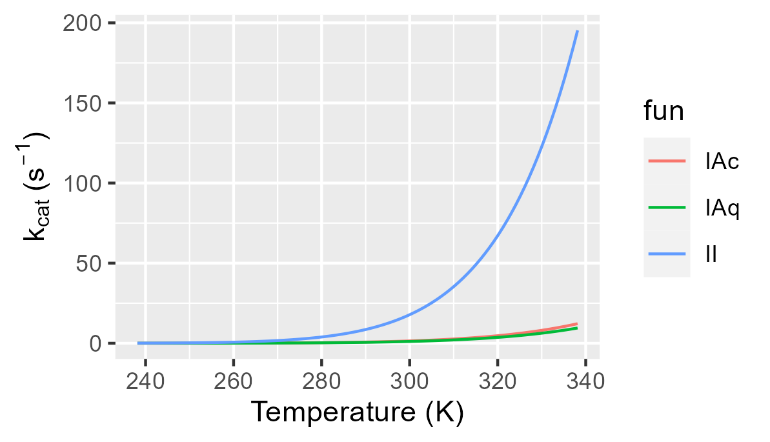

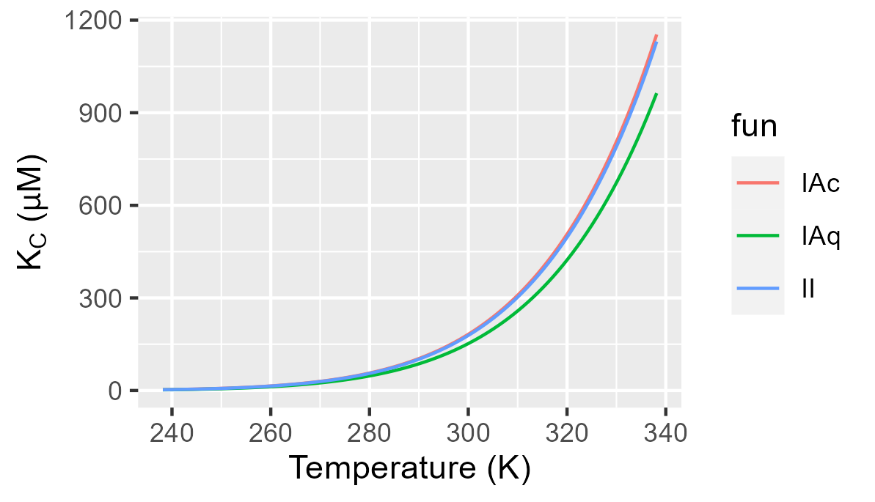

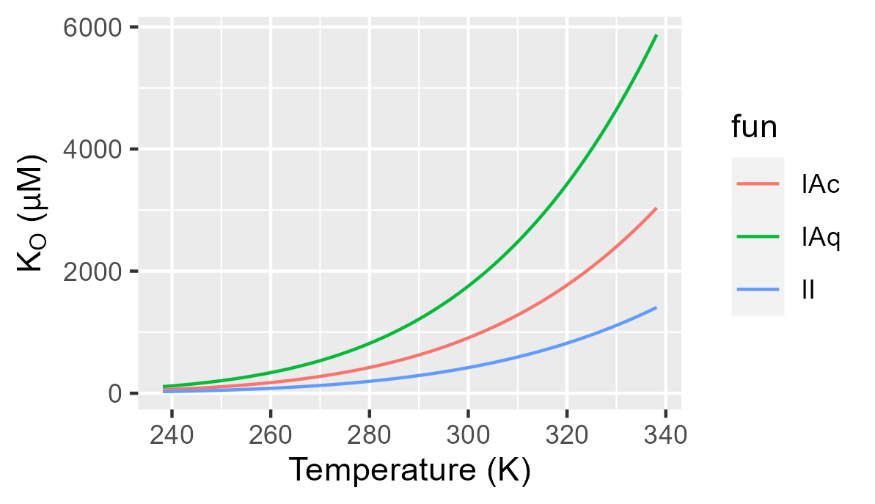

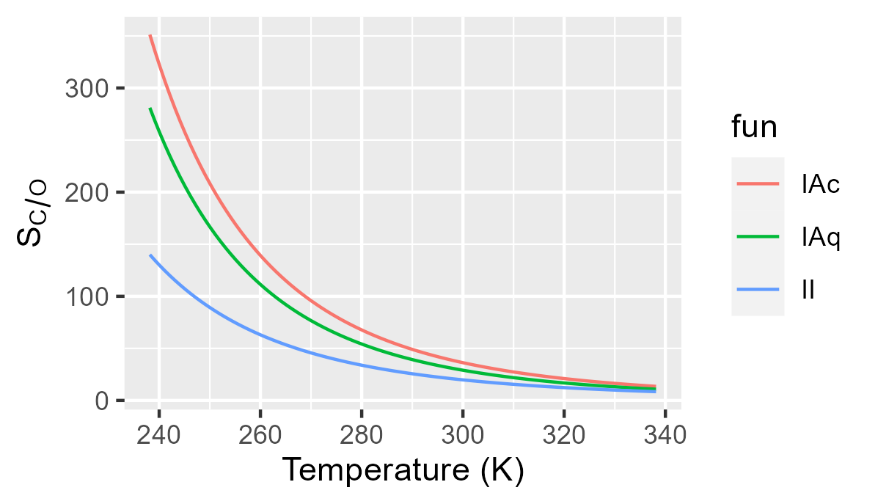


Fig. S8: Graphs of temperature dependence for each parameter for Rubisco forms.

Modeled temperature dependence of kinetics of form II (II), form IAq (IAq), and form IAc (IAc). A) *k*_cat,c_, B) K_C_, C) K_O_, D) S_C/O_


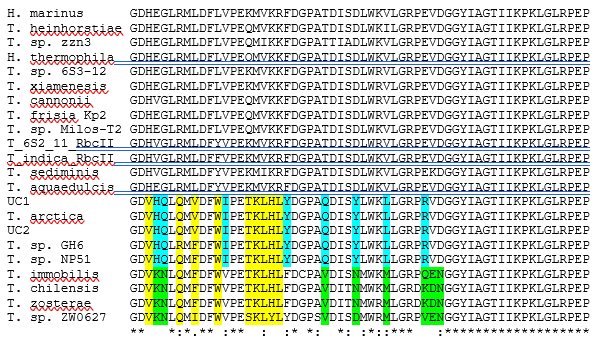

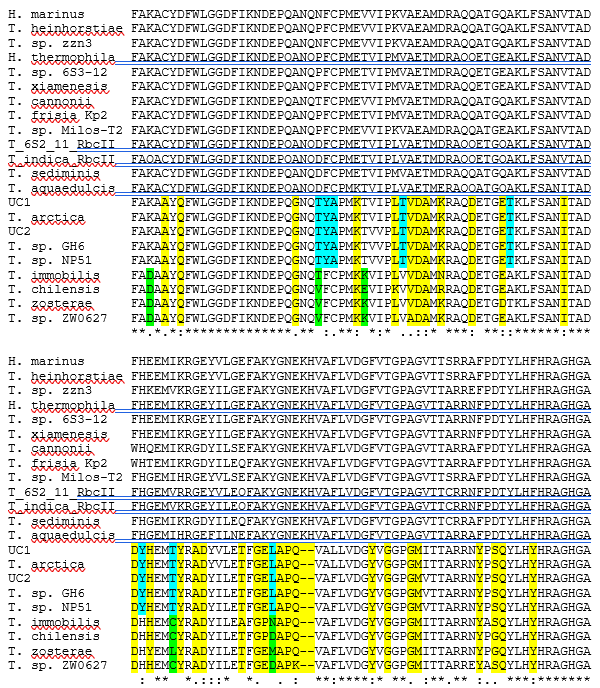

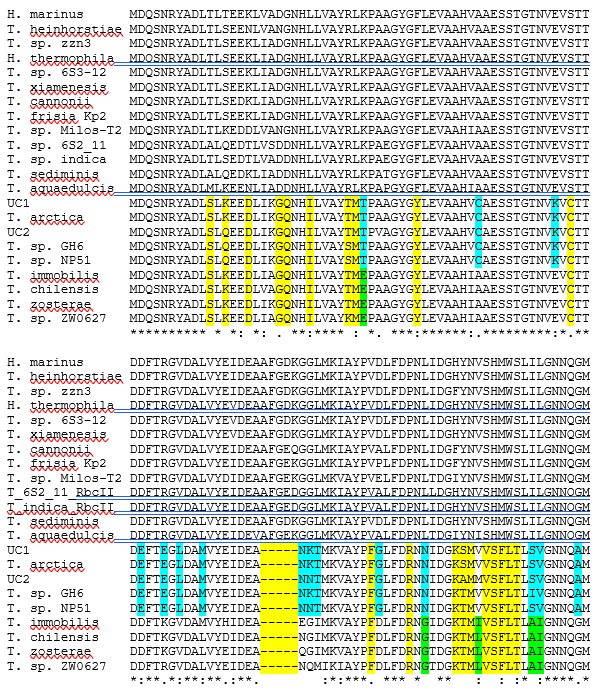

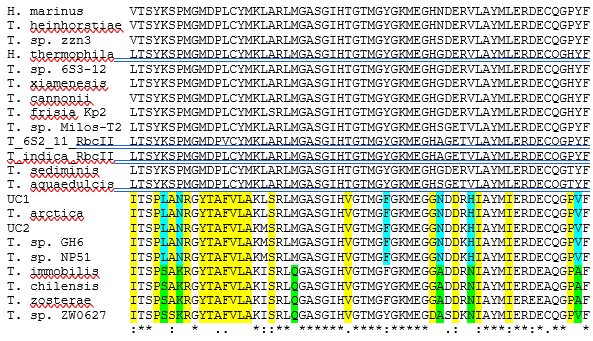

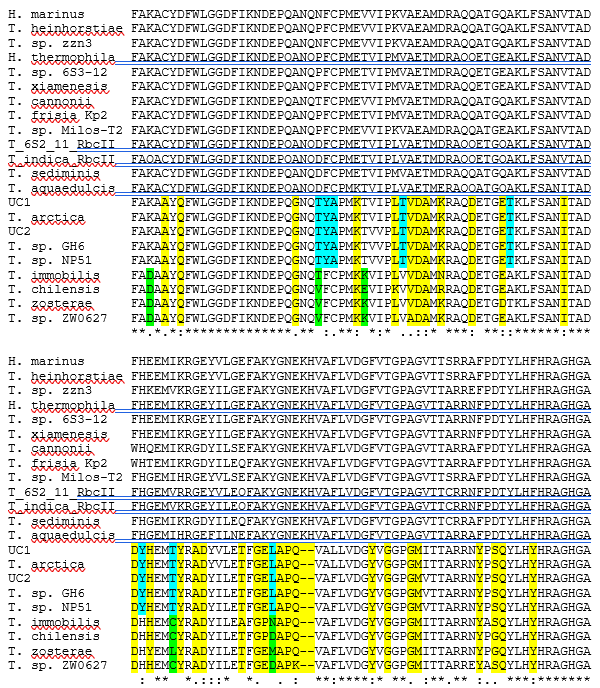

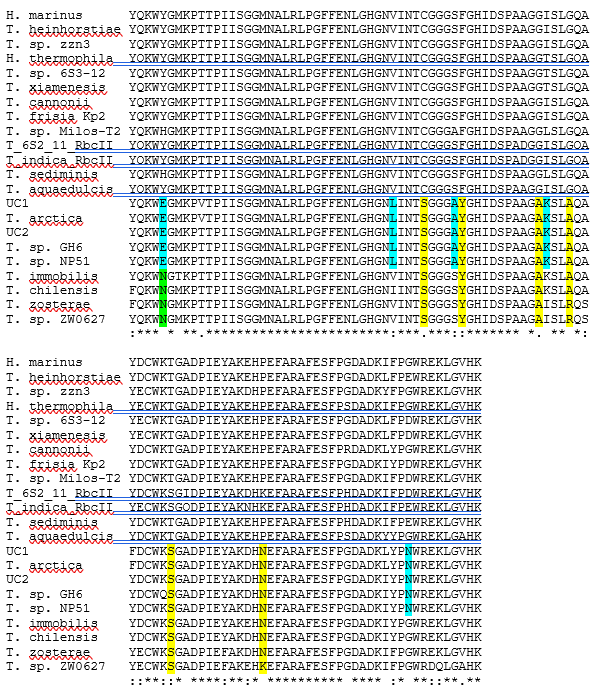


300

60

466

420

360

240

180

120

Fig. S9: Rubisco Form II alignment & mutations.

Rubisco Form II sequences aligned in CLUSTAL format. Where all cold-adapted species differ from overall consensus, amino acids are highlighted yellow. When the cold-adapted species disagree and are different from the overall consensus, those from Clade 2 are highlighted blue and otherwise are highlighted green. Consensus symbols under sequences: ***** = all agree, **:** = all changes conservative, **.** = all changes semi-conservative, empty = not conserved.


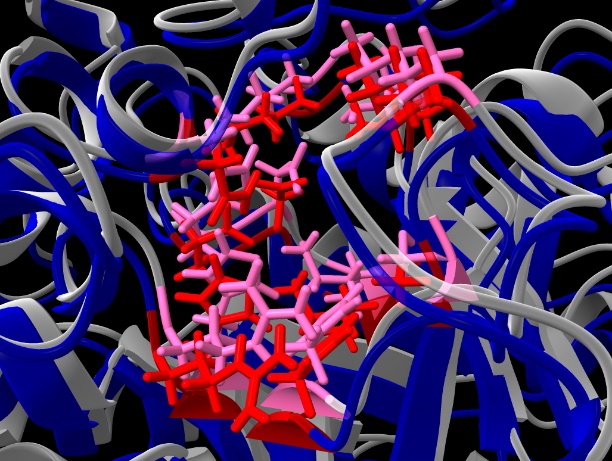

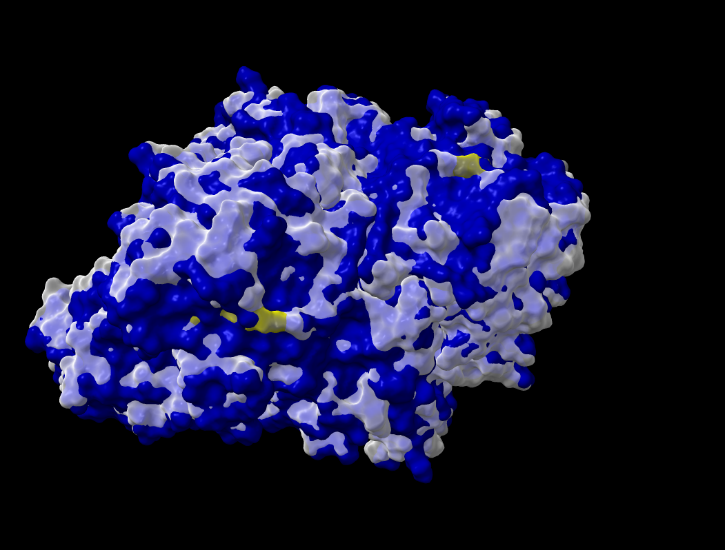

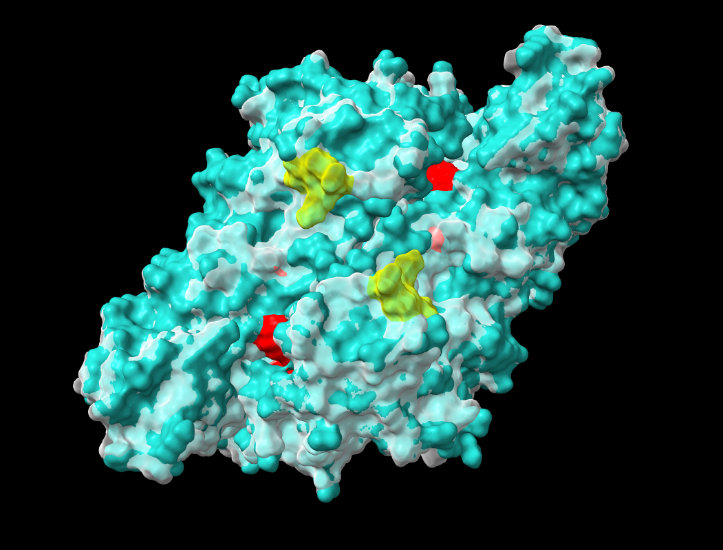

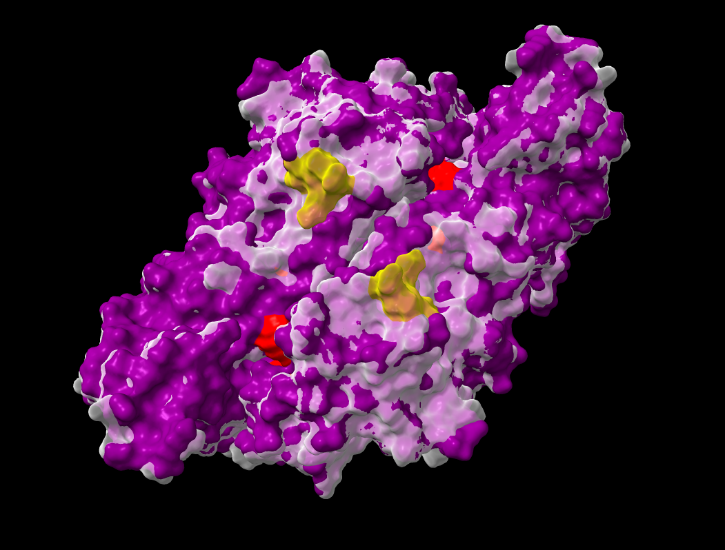

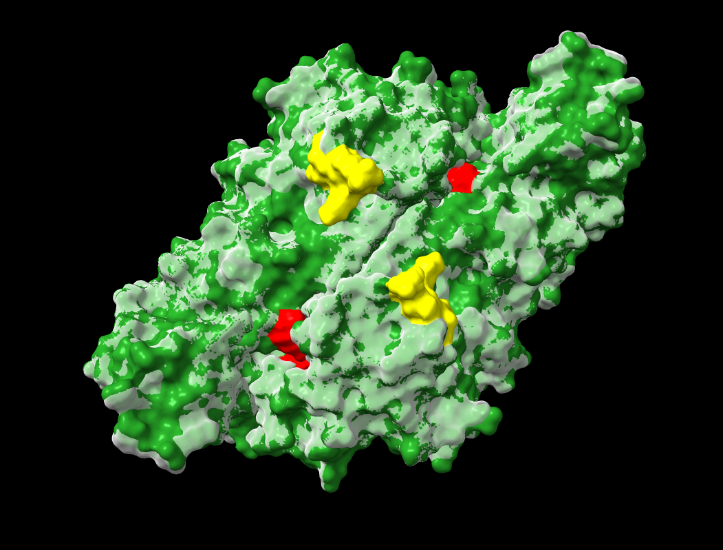

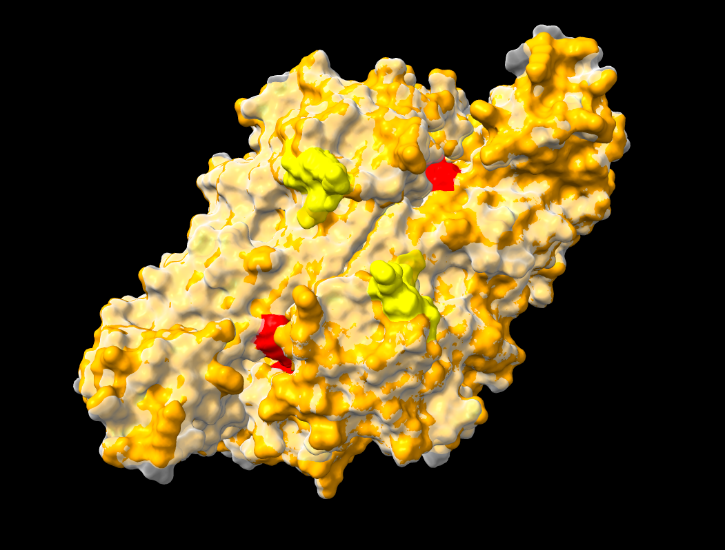

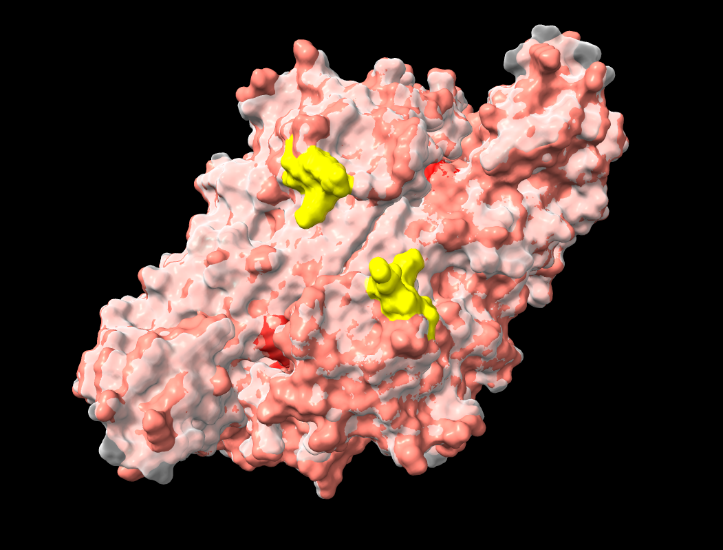


**G**

**F**

**E**

**D**

**C**

**B**

**A**

Fig. S10: Rubisco form II additional alignments.

Supplemental views of RbcII alignment, visualized as in Fig. 5. A) *H. marinus* form II (transparent white) and UC1 form II (blue) active sites. Pink is *H. marinus* atoms, while red is UC1 atoms. B) *H. marinus* form II (transparent white) and UC1 form II (blue) protein back. *H. marinus* loop amino acids absent in *T. arctica* UC1 colored yellow. C–G) *H. marinus* form II (transparent white) compared to form II structures of other *Thiomicrorhabdus* species (opaque structures). Protrusion amino acids colored yellow if present. Active sites colored red in opaque structures. C–D) Sequences with signals of cold adaptation: C) *T.* sp. NP51 (cyan), D) *T. chilensis* (purple). E–G) Sequences with no thermal adaptation signal: E) *T. frisia* Kp2 (green), F) *T. indica* (orange), G) *T. aquaedulcis* (pink).

Table S1: Autotrophic marker genes

| **Pathway** | **Gene** | **Abbreviation** |
| --- | --- | --- |
| Calvin-Benson-Bassham (CBB) | Rubisco large subunit [E.C. 4.1.1.39/KO 01601] | CBBa |
| Wood-Ljungdahl (W-L) | Acetyl-CoA synthase [E.C. 2.3.1.169/KO 14138] | W-L |
| 4-hydroxybutyrate/dicarboxylate cycle (4-HB/DC) | 4-hydroxybutyryl-CoA dehydratase [E.C./KO 14534] | 4-HB |
| 3-hydroxyproprionate/4-hydroxybutyrate cycle  (3-HP/4-HB) |  |  |
| 3-hydroxyproprionate bicycle (3-HP) | 2-methylfumaryl-CoA isomerase [E.C. 5.4.1.3/KO 14470] | 3-HP |
| Reductive TCA cycle (rTCA) | Citryl-CoA synthase [E.C. 6.2.1.18/KO 15232] | rTCA^1^ |
|  | ATP-citrate lyase α [E.C. 2.3.3.8/KO 15230] |  |

^1^The abundance of both marker genes for the rTCA cycle was combined, as the two enzymes substitute for each other in the pathway.

Table S2: Thiomicrorhabdus genome completeness, contamination, sampling conditions, and temperature and salinity growth ranges. Completeness and contamination calculated by CheckM (2). NF = not found, N/A = MAG genomes’ temperature and salinity growth ranges.

| **Species** | **Genome Type** | **Completeness** | **Contamination** | **In-Situ Temp. (°C)** | **In-Situ Salinity** | **Minimum Temp. (°C)** | **Optimum Temp. (°C)** | **Maximum Temp. (°C)** | **Minimum Sal.** | **Optimum Sal.** | **Maximum Sal.** | **Environment** | **Comment** | **Source** | **NCBI GenBank Number** |
| --- | --- | --- | --- | --- | --- | --- | --- | --- | --- | --- | --- | --- | --- | --- | --- |
| UC1 | MAG | 99.31 | 0.61 | -6 | 109-140 ppt | N/A | N/A | N/A | N/A | N/A | N/A | Cryopeg brine |  | This study | TBD |
| UC2 | MAG | 98.60 | 0.00 | -6 | 109-140 ppt | N/A | N/A | N/A | N/A | N/A | N/A | Cryopeg brine |  | This study | TBD |
| *T. arctica* | Isolate | 99.66 | 0.00 | 0 | NF | -2 | 11.5-13.2 | 20.8 | 40 mM NaCl | 250 mM NaCl | 1200 mM NaCl | Marine arctic sediment |  | (3) | GCA_000381085.1 |
| *T. aquaedulcis* | Isolate | 98.63 | 0.23 | 15 | <40 mM Cl^-^ | 0 | 22 | 25 | NF | 220-320 mM Na+ | 520 mM Na^+^ | Meromictic lake water |  | (4, 5) | GCA_004001325.1 |
| *T. cannonii* | Isolate | 99.93 | 0.62 | 24.2 | 13.3 mg/L | 15 | 32 | 35 | 80 mM NaCl | 80 mM NaCl | 517 mM NaCl | Anchialine sinkhole chemocline water |  | (6) | GCA_013391695.1 |
| *T. chilensis* | Isolate | 100.00 | 0.00 | NF | NF | 3.5 | 32-37 | 42 | 100 mM NaCl | 470 mM NaCl | 1240 mM NaCl | Continental shelf sediment |  | (7) | GCA_000483485.1 |
| *T. frisia* Kp2 | Isolate | 100.00 | 0.00 | NF | NF | 3.5 | 32-35 | 39 | 100 mM Na^+^ | 470 mM Na^+^ | 1240 mM Na^+^ | Hydrothermal vent |  | (8, 9) | GCA_00478585.1 |
| *T. heinhorstiae* | Isolate | 99.66 | 1.10 | 24.2 | 13.3 mg/L | 15 | 32.8 | 35 | 80 mM NaCl | 400 mM NaCl | 689 mM NaCl | Anchialine sinkhole chemocline water |  | (6) | GCA_013391765.1 |
| *T. immobilis* (Am-19) | Isolate | 99.89 | 0.21 | NF | NF | 5 | 30 | 37 | 0 mM NaCl | Unknown | 1300 mM NaCl | Brackish lake sediment |  | (10) | GCA_021654855.1 |
| *T. indica* | Isolate | 99.25 | 0.29 | NF | NF | 10 | 28 | 45 | 85 | 680 mM NaCl | 1700 | Northwest Indian Ocean seawater near hydrothermal vent | No info on distance to nearest vent | (11) | GCA_004293625.1 |
| *T. lithotrophica* (XGS_01) | Isolate | 100 | 0.00 | NF | NF | 4 | 30 | 45 | 170 mM NaCl | 510 mM NaCl | 850 mM NaCl | Coastal sediment of Guanyinshan beach, Xiamen, China |  | (12) | GCA_029201445.1 |
| *T. marina* (6S2-11) | Isolate | 99.09 | 0.00 | NF | NF | 20 | 35 | 37 | .5% (w/v) NaCl | 2.5% (w/v) NaCl | 5% (w/v) NaCl | Weihei, China tidal zone sediment | Renamed *T. marina* after download | (13) | GCA_017571465.1 |
| *T. sediminis* | Isolate | 99.66 | 0.00 | NF | NF | 10 | 30 | 40 | 85 | 510 mM NaCl | 1530 | Coastal sediment |  | (14) | GCA_005885815.1 |
| *T. xiamenesis* | Isolate | 99.25 | 0.38 | NF | NF | 4 | 28 | 45 | 85 | 340 mM NaCl | 1530 | Coastal sediment |  | (14) | GCA_013282625.1 |
| *T. zosterae (Thiosulfati-vibrio zosterae)* | Isolate | 98.17 | 0.17 | NF | NF | 5 | 22 | 37 | 0% | 2% (w/v) NaCl | 5% | Brackish lake eelgrass | Classified as *Thiosulfativibrio* after download | (15) | GCA_011398155.1 |
| *T.* sp. 354-166_108 | MAG | 94.17 | 3.80 | NF | NF | N/A | N/A | N/A | N/A | N/A | N/A | Mid-Atlantic Ridge Rainbow hydrothermal fluid |  | (16) | GCA_026989175.1 |
| *T.* sp. 355-202_026 | MAG | 78.58 | 1.39 | NF | NF | N/A | N/A | N/A | N/A | N/A | N/A | Mid-Atlantic Ridge Rainbow hydrothermal fluid |  | (16) | GCA_026988215.1 |
| *T.* sp. 4281-140_065VB | MAG | 92.69 | 7.21 | NF | NF | N/A | N/A | N/A | N/A | N/A | N/A | East Pacific Rise Bio9 hydrothermal fluid |  | (16) | GCA_027068835.1 |
| *T.* sp. 6S3-12 | Isolate | 99.25 | 0.38 | NF | NF | NF | NF | NF | NF | NF | NF | Weihei, China coastal sediment | Same authors as *T. marina* | NCBI | GCA_017571475.1 |
| *T.* sp. A3_174 | MAG | 94.61 | 3.55 | See comment | NF | N/A | N/A | N/A | N/A | N/A | N/A | Eastern Lau Spreading Center ABE | Vent fluid 297-317°C in 2009 (17) | (16) | GCA_027031285.1 |
| *T.* sp. D96SM_15 | MAG | 78.19 | 2.42 | See comment | NF | N/A | N/A | N/A | N/A | N/A | N/A | Sulfide sample from active hydrothermal chimney | Vent fluid 362°C, samples taken 10cm away from vent with seepage observed | (18) | GCA_021648625.1 |
| *T.* sp. D96SM_37 | MAG | 55.70 | 1.17 | See comment | NF | N/A | N/A | N/A | N/A | N/A | N/A | Sulfide sample from active hydrothermal chimney | Vent fluid 362°C, samples taken 10cm away from vent with seepage observed | (18) | GCA_021648185.1 |
| *T.* sp. D100SM_12 | MAG | 71.18 | 1.19 | See comment | NF | N/A | N/A | N/A | N/A | N/A | N/A | Sulfide sample from active hydrothermal chimney | Vent fluid 365°C, samples taken 10cm away from vent with seepage observed | (18) | GCA_021647665.1 |
| *T.* sp. GH6 | MAG | 75.41 | 3.53 | NF | NF | NF | NF | NF | NF | NF | NF | Cold saline spring, Nunavut, Canada |  | NCBI | GCA_027858195.1 |
| *T.* sp. M17_146 | MAG | 93.24 | 4.02 | See comment | NF | N/A | N/A | N/A | N/A | N/A | N/A | Eastern Lau Spreading Center Mariner | Vent fluid 338-359°C in 2009 (18) | (16) | GCA_026996215.1 |
| *T.* sp. M17_171 | MAG | 84.92 | 5.03 | See comment | NF | N/A | N/A | N/A | N/A | N/A | N/A | Eastern Lau Spreading Center Mariner | Vent fluid 338-359°C in 2009 (18) | (16) | GCA_027081435.1 |
| *T.* sp. Milos-T2 | Isolate | 100.00 | 0.00 | 25-60 | 39-58 ppt | NF | NF | NF | NF | NF | NF | Milos, Greece shallow water hydrothermal vent |  | (19) | GCA_000702325.1 |
| *T.* sp. NP51 | MAG | 93.93 | 2.48 | -1.3 - 7 | ~8% | N/A | N/A | N/A | N/A | N/A | N/A | Bacterial streamers in cold saline spring |  | (20) | GCA_006222135.1 |
| *T.* sp. S012_012 | MAG | 76.25 | 4.51 | See comment | 40.55 g/kg* | N/A | N/A | N/A | N/A | N/A | N/A | Brothers Volcano Upper Caldera sediment | Vent fluid 276.5°C (21) | (16) | GCA_027071695.1 |
| *T.* sp. S012_68 | MAG | 93.51 | 3.20 | See comment | 40.55 g/kg* | N/A | N/A | N/A | N/A | N/A | N/A | Brothers Volcano Upper Caldera sediment | Vent fluid 276.5°C | (21) | GCA_015487495.1 |
| *T.* sp. S012_085 | MAG | 97.83 | 0.91 | See comment | 40.55 g/kg* | N/A | N/A | N/A | N/A | N/A | N/A | Brothers Volcano Upper Caldera sediment | Vent fluid 276.5°C (21) | (16) | GCA_027072875.1 |
| *T.* sp. S012_107 | MAG | 57.39 | 1.56 | See comment | 40.55 g/kg* | N/A | N/A | N/A | N/A | N/A | N/A | Brothers Volcano Upper Caldera sediment | Vent fluid 276.5°C (21) | (16) | GCA_027072725.1 |
| *T.* sp. S012_112 | MAG | 83.16 | 3.30 | See comment | 40.55 g/kg* | N/A | N/A | N/A | N/A | N/A | N/A | Brothers Volcano Upper Caldera sediment | Vent fluid 276.5°C | (21) | GCA_015488565.1 |
| *T.* sp. S013_018 | MAG | 81.97 | 2.80 | See comment | 34.42 g/kg* | N/A | N/A | N/A | N/A | N/A | N/A | Brothers Volcano NW Caldera A sediment | Vent fluid 318°C (21) | (16) | GCA_027074675.1 |
| *T.* sp. S013_057VB | MAG | 51.64 | 5.98 | See comment | 34.42 g/kg* | N/A | N/A | N/A | N/A | N/A | N/A | Brothers Volcano NW Caldera A sediment | Vent fluid 318°C (21) | (16) | GCA_027008475.1 |
| *T.* sp. S013_67 | MAG | 90.75 | 7.59 | See comment | 34.42 g/kg* | N/A | N/A | N/A | N/A | N/A | N/A | Brothers Volcano NW Caldera A sediment | Vent fluid 318°C | (21) | GCA_015487015.1 |
| *T.* sp. S139_146 | MAG | 86.34 | 3.79 | See comment | 40.55 g/kg* | N/A | N/A | N/A | N/A | N/A | N/A | Brothers Volcano Upper Caldera sediment | Vent fluid 276.5°C (21) | (16) | GCA_027076365.1 |
| *T.* sp. S139_169 | MAG | 73.6 | 2.60 | See comment | 40.55 g/kg* | N/A | N/A | N/A | N/A | N/A | N/A | Brothers Volcano Upper Caldera sediment | Vent fluid 276.5°C (21) | (16) | GCA_027078285.1 |
| *T.* sp. S140_061 | MAG | 87.59 | 6.39 | See comment | 36.26 g/kg* | N/A | N/A | N/A | N/A | N/A | N/A | Brothers Volcano NW Caldera B sediment | Vent fluid 303°C (21) | (16) | GCA_026999805.1 |
| *T.* sp. S140_168 | MAG | 77.18 | 7.68 | See comment | 36.26 g/kg* | N/A | N/A | N/A | N/A | N/A | N/A | Brothers Volcano NW Caldera B sediment | Vent fluid 303°C (21) | (16) | GCA_026998755.1 |
| *T.* sp. S140_198 | MAG | 82.99 | 1.10 | See comment | 36.26 g/kg* | N/A | N/A | N/A | N/A | N/A | N/A | Brothers Volcano NW Caldera B sediment | Vent fluid 303°C (21) | (16) | GCA_026999805.1 |
| *T.* sp. S141_101 | MAG | 98.52 | 0.46 | See comment | 36.26 g/kg* | N/A | N/A | N/A | N/A | N/A | N/A | Brothers Volcano NW Caldera B sediment | Vent fluid 318°C (21) | (16) | GCA_027051335.1 |
| *T.* sp. S141_143 | MAG | 67.26 | 1.99 | See comment | 36.26 g/kg* | N/A | N/A | N/A | N/A | N/A | N/A | Brothers Volcano NW Caldera B sediment | Vent fluid 318°C (21) | (16) | GCA_027046185.1 |
| *T.* sp. S141_227 | MAG | 90.09 | 2.06 | See comment | 36.26 g/kg* | N/A | N/A | N/A | N/A | N/A | N/A | Brothers Volcano NW Caldera B sediment | Vent fluid 318°C (21) | (16) | GCA_027045705.1 |
| *T.* sp. UWMA-0242 | MAG | 66.95 | 1.14 | NF | NF | N/A | N/A | N/A | N/A | N/A | N/A | Cayman Rise Shallow rising plume |  | (22) | GCA_012961475.1 |
| *T.* sp. zzn3 | Isolate | 99.54 | 0.24 | NF | NF | NF | NF | NF | NF | NF | NF | Sediment in Zhangzhou, China |  | NCBI | GCA_029767755.1 |
| *T.* sp. ZW0627 | Isolate | 100 | 0.92 | NF | NF | NF | NF | NF | NF | NF | NF | Sediment in Zhangzhou, China |  | NCBI | GCA_029767005.1 |

*Salinity calculated from measurements of major seawater constituent concentrations (Na, Ca, K, Cl, Mg, SO_4_)

Table S3: Modeled temperature dependence equations for each kinetic parameter

| Parameter | RbcIAq | RbcIAc | RbcII |
| --- | --- | --- | --- |
| *k*_cat,C_ | $e^{19.05-\frac{47.2}{.00831*T}}$ | $e^{19.83-\frac{47.2}{.00831*T}}$ | $e^{24.10-\frac{52.9}{.00831*T}}$ |
| K_C_ | $e^{21.39-\frac{40.8}{.00831*T}}$ | $e^{21.57-\frac{40.8}{.00831*T}}$ | $e^{21.55-\frac{40.8}{.00831*T}}$ |
| K_O_ | $e^{18.18-\frac{26.7}{.00831*T}}$ | $e^{17.52-\frac{26.7}{.00831*T}}$ | $e^{16.75-\frac{26.7}{.00831*T}}$ |
| S_C/O_ | $e^{-5.377+\frac{21.8}{.00831*T}}$ | $e^{-5.154+\frac{21.8}{.00831*T}}$ | $e^{-4.558+\frac{18.8}{.00831*T}}$ |

Table S4: Source of ΔH values for kinetic parameter temperature dependence equations

| Parameter | RbcIA | | | RbcII | | |
| --- | --- | --- | --- | --- | --- | --- |
|  | ΔH (kJ/mol) | Source | Ref | ΔH (kJ/mol) | Source | Ref |
| *k*_cat,C_ | 47.2 | Average of 3 of the 4 reported proteobacteria values (*Thiomicrospira (Tms.) thyasira* may have both form IA and form II enzymes, as its closest relative *Tms. pelophila* does (23)) | (24) | 52.9 | Weighted average of available values^1^ | (25) |
| K_C_ | 40.8 | Weighted average of available values^1^ | (25) | 40.8 | Weighted average of available values^1^ | (25) |
| K_O_ | 26.7 | Average of all in-vivo K_O­_ ΔH^2^ | (25) | 26.7 | Average of all in-vivo K_O­_ ΔH^2^ | (25) |
| S_C/O_ | -21.8 | Weighted average of available non-Proteobacteria values^1^ | (25) | -18.8 | Value from *R. rubrum* form II | (25) |

^1^Excluding Rhodophyta, as Galmés et al. 2016 (25) notes how different it is from the others

^2^Excluding *A. thaliana* because its r^2^ value was much lower than the others

Table S5: Known values of kinetic parameters for Rubisco forms

| Parameter | RbcIAq | | | RbcIAc | | | RbcII | | |
| --- | --- | --- | --- | --- | --- | --- | --- | --- | --- |
|  | Known Value | Temperature (°C) | Ref | Value | Temperature (°C) | Ref | Value | Temperature (°C) | Ref |
| *k*_cat,C_ | 1.375^1^ @ 30 | 30 | (26) | 2.980^1^ | 30 | (26) | 15.6 | 25 | (27) |
| K_C_ | 138 @ 25 | 25 | (28)^2^ | 165 | 25 | (28)^3^ | 162 | 25 | (27) |
| K_O_ | 1640 @ 25 | 25 | (28)^2^ | 845 | 25 | (28)^3^ | 394 | 25 | (28)^4^ |
| S_C/O_ | 26.6 @ 30 | 30 | (26) | 33.1 | 30 | (26) | 20.7 | 25 | (27) |

^1^Adjusted from specific activity (umol CO_2_ fixed * min^-1^ * mg Rbc^-1^) using conversion factor of 1.146 * specific activity = *k_cat_* (see below)

^2^Values from *Thiobacillus denitrificans*, as it is the only species with only Form IAq and not Form IAc with reported kinetics

^3^Avg. of all non-*T. denitrificans* form IA species (average values for each species were calculated first)

^4^Avg. of all Form II species (average values for each species were calculated first)

Specific activity conversion factor derivation:

$$\frac{1 \mu mol CO_{2} fixed}{min*mg Rbc}*\frac{1 min}{60 sec}*\frac{1 mol CO_{2}}{{10}^{6} \mu mol CO_{2}}*\frac{1000 mg Rbc}{1 g Rbc}*\frac{550000 g Rbc}{1 mol Rbc}*\frac{1 mol Rbc}{8 mol sites}=\frac{1.146 mol CO_{2}}{sec*mol sites}=\frac{1.146 rxns}{sec}$$

Table S6: Modeled carboxylation rate equations for Rubisco forms

| Model | Gross | Canonical tradeoff |
| --- | --- | --- |
| Equation | $k_{cat,C}*\frac{\left[ CO_{2} \right]}{\left[ CO_{2} \right]+K_{C}+K_{C}*\frac{\left[ O_{2} \right]}{K_{O}}}$ | $k_{cat,C}*\frac{\left[ CO_{2} \right]}{\left[ CO_{2} \right]+K_{C}+K_{C}*\frac{\left[ O_{2} \right]}{K_{O}}}*\left( 1-\frac{\left[ O_{2} \right]}{2*S_{C/O}*\left[ CO_{2} \right]} \right)$ |
| RbcIAq | $e^{19.05-\frac{47.2}{.00831*T}}*\frac{\left[ CO_{2} \right]}{\left[ CO_{2} \right]+e^{21.39-\frac{40.8}{.00831*T}}+e^{3.21-\frac{14.1}{.00831*T}}\left[ O_{2} \right]}$ | $e^{19.05-\frac{47.2}{.00831*T}}*\frac{\left[ CO_{2} \right]}{\left[ CO_{2} \right]+e^{21.39-\frac{40.8}{.00831*T}}+e^{3.21-\frac{14.1}{.00831*T}}\left[ O_{2} \right]}*(1-\frac{\left[ O_{2} \right]}{2*e^{-5.377+\frac{21.8}{.00831*T}}*\left[ CO_{2} \right]})$ |
| RbcIAc | $e^{19.83-\frac{47.2}{.00831*T}}*\frac{\left[ CO_{2} \right]}{\left[ CO_{2} \right]+e^{21.57-\frac{40.8}{.00831*T}}+e^{4.05-\frac{14.1}{.00831*T}}\left[ O_{2} \right]}$ | $e^{19.83-\frac{47.2}{.00831*T}}*\frac{\left[ CO_{2} \right]}{\left[ CO_{2} \right]+e^{21.57-\frac{40.8}{.00831*T}}+e^{4.05-\frac{14.1}{.00831*T}}\left[ O_{2} \right]}*(1-\frac{\left[ O_{2} \right]}{2*e^{-5.154+\frac{21.8}{.00831*T}}*\left[ CO_{2} \right]})$ |
| RbcII | $e^{24.1-\frac{52.9}{.00831*T}}*\frac{\left[ CO_{2} \right]}{\left[ CO_{2} \right]+e^{21.55-\frac{40.8}{.00831*T}}+e^{4.8-\frac{14.1}{.00831*T}}\left[ O_{2} \right]}$ | $e^{24.1-\frac{52.9}{.00831*T}}*\frac{\left[ CO_{2} \right]}{\left[ CO_{2} \right]+e^{21.55-\frac{40.8}{.00831*T}}+e^{4.8-\frac{14.1}{.00831*T}}\left[ O_{2} \right]}*(1-\frac{\left[ O_{2} \right]}{2*e^{-4.558+\frac{18.8}{.00831*T}}*\left[ CO_{2} \right]})$ |

Table S7: Mesophile reference Rubisco sequences for thermal adaptation analysis

| **Rubisco Form** | **Reference Genomes** |
| --- | --- |
| IAc | *H. marinus, T. cannonii, T. heinhorstiae, T. marina, T. sediminis* |
| IAq | *H. marinus, T. cannonii, T. heinhorstiae* |
| II | *H. marinus, T. cannonii, T. heinhorstiae, T. marina, T. sediminis* |

Table S8: Scores of thermal adaptation index. Bolded scores met criteria for thermal adaptation index. — = not found in genome

| **Genome** | **Form IAc** | | | **Form IAq** | | | **Form II** | | |
| --- | --- | --- | --- | --- | --- | --- | --- | --- | --- |
|  | *# cold* | *# hot* | *Score* | *# cold* | *# hot* | *Score* | *# cold* | *# hot* | *Score* |
| *H. thermophila* | 0 | 2 | –2 | 1 | 2 | –1 | 2 | 0 | 2 |
| *T. aquaedulcis* | — | — | — | 3 | 2 | 1 | 1 | 1 | 0 |
| *T. arctica* | — | — | — | 1 | 2 | –1 | 7 | 2 | **5^c^** |
| *T. arctica* UC1 | — | — | — | 1 | 2 | –1 | 6 | 2 | **4^c^** |
| *T. chilensis* | 1 | 2 | –1 | 2 | 4 | –2 | 5 | 0 | **5^c^** |
| *T. frisia* Kp2 | 0 | 2 | –2 | 2 | 2 | 0 | 0 | 1 | –1 |
| *T. immobilis* | 2 | 2 | 0 | 3 | 2 | 1 | 6 | 1 | **5** |
| *T. indica* | 1 | 5 | **–4^h^** | — | — | — | 1 | 0 | 1 |
| *T. lithotrophica* | 1 | 4 | **–3^h^** | — | — | — | — | — | — |
| *T.* sp. 6S3-12 | 2 | 0 | 2 | 2 | 0 | 2 | 3 | 2 | 1 |
| *T.* sp. D96SM | 0 | 4 | **–4^h^** | — | — | — | — | — | — |
| *T.* sp. Milos-T2 | — | — | — | 1 | 1 | 0 | 3 | 2 | 1 |
| *T.* sp. NP51 | — | — | — | — | — | — | 6 | 1 | **5^c^** |
| *T.* sp. S012-112 | 0 | 5 | **–5^h^** | — | — | — | — | — | — |
| *T.* sp. S141-101 | 1 | 4 | **–3^h^** | — | — | — | — | — | — |
| *T.* sp. S141-227 | 0 | 5 | **–5^h^** | — | — | — | — | — | — |
| *T.* sp. UC2 | — | — | — | — | — | — | 5 | 1 | **4^c^** |
| *T.* sp. ZW0627 | 2 | 0 | 2 | 2 | 3 | –1 | 6 | 2 | **4^c^** |
| *T.* sp. zzn3 | 2 | 0 | 2 | 3 | 3 | 0 | 2 | 1 | 1 |
| *T. xiamenesis* | 2 | 0 | 2 | 2 | 0 | 2 | 3 | 2 | 1 |
| *T. zosterae* | 2 | 4 | –2 | 0 | 2 | –2 | 4 | 0 | **4^c^** |

^h^Hot-adapted

^c^Cold-adapted

Table S9: Cryopeg and sea ice sample environments. Data collected from Rapp et al. 2021 (29).

| **Name** | **Sample Type** | **Temperature (°C)** | **Salinity (ppt)** | **Comments** |
| --- | --- | --- | --- | --- |
| CBIW_2017 | Cryopeg brine | -6 | 140 | CBIW borehole sampled in 2017 |
| CBIW_2018 | Cryopeg brine | -6 | 121 | CBIW borehole sampled in 2018 |
| CBIA | Cryopeg brine | -6 | 112 | CBIA borehole sample |
| CB1 | Cryopeg brine | -6 | 122 | CB1 borehole sample |
| CB4 | Cryopeg brine | -6 | 121 | CB4 borehole sample |
| SB_2017 | Sea ice brine | -4 | 78 | Sea ice brine sampled in 2017 |
| SB_2018 | Sea ice brine | -3 | 75 | Sea ice brine sampled in 2018 |
| SI3U | Sea ice section | -5 | 18 | Upper third of sea ice core |
| SI3L | Sea ice section | -3 | 18 | Lower third of sea ice core |

Table S10: Direction of change of thermal adaptation indices. Direction of change for index (increase (+) or decrease (-)) indicative of either hot or cold adaptation. Table adapted from Raymond-Bouchard et al. 2021 (30).

| **Index** | **Cold-Adapted** | **Hot-Adapted** |
| --- | --- | --- |
| # Acidic Residues (D,E) | - | + |
| # Charged Residues (D,E,H,R,K) | - | + |
| # Polar Residues (C,N,Q,S,T,Y) | + | - |
| # Proline Residues (P) | - | + |
| # Glycine Residues (G) | + | - |
| # Serine Residues (S) | + | - |
| Aromaticity | - | + |
| Aliphatic Index | - | + |
| Hydrophobicity (using GRAVY) | - | + |
| Arginine/Lysine Ratio (R/K) | - | + |

**Supplemental References:**

1. Laing WA, Ogren WL, Hageman RH. 1974. Regulation of Soybean Net Photosynthetic CO2 Fixation by the Interaction of CO2, O2, and Ribulose 1,5-Diphosphate Carboxylase 1 2. Plant Physiol 54:678–685.

2. Parks DH, Imelfort M, Skennerton CT, Hugenholtz P, Tyson GW. 2015. CheckM: assessing the quality of microbial genomes recovered from isolates, single cells, and metagenomes. Genome Res 25:1043–1055.

3. Knittel K, Kuever J, Meyerdierks A, Meinke R, Amann R, Brinkhoff T. 2005. Thiomicrospira arctica sp. nov. and Thiomicrospira psychrophila sp. nov., psychrophilic, obligately chemolithoautotrophic, sulfur-oxidizing bacteria isolated from marine Arctic sediments. Int J Syst Evol Micr 55:781–786.

4. Kojima H, Fukui M. 2019. Thiomicrorhabdus aquaedulcis sp. nov., a sulfur-oxidizing bacterium isolated from lake water. Int J Syst Evol Micr 69:2849–2853.

5. Kubo K, Kojima H, Fukui M. 2014. Vertical distribution of major sulfate-reducing bacteria in a shallow eutrophic meromictic lake. Syst Appl Microbiol 37:510–519.

6. Updegraff T, Schiff-Clark G, Gossett H, Parsi S, Peterson R, Whittaker R, Dennison C, Davis M, Bray J, Boden R, Scott K. 2022. Thiomicrorhabdus heinhorstiae sp. nov. and Thiomicrorhabdus cannonii sp. nov.: novel sulphur-oxidizing chemolithoautotrophs isolated from the chemocline of Hospital Hole, an anchialine sinkhole in Spring Hill, Florida, USA. Int J Syst Evol Micr 72.

7. Brinkhoff T, Muyzer G, Wirsen CO, Kuever J. 1999. Thiomicrospira chilensis sp. nov., a mesophilic obligately chemolithoautotrophic sulfur-oxidizing bacterium isolated from a Thioploca mat. Int J Syst Evol Micr 49:875–879.

8. Brinkhoff T, Muyzer G, Wirsen CO, Kuever J. 1999. Thiomicrospira kuenenii sp. nov. and Thiomicrospira frisia sp. nov., two mesophilic obligately chemolithoautotrophic sulfur-oxidizing bacteria isolated from an intertidal mud flat. Int J Syst Evol Micr 49:385–392.

9. Boden R, Scott KM, Williams J, Russel S, Antonen K, Rae AW, Hutt LP. 2017. An evaluation of Thiomicrospira, Hydrogenovibrio and Thioalkalimicrobium: reclassification of four species of Thiomicrospira to each Thiomicrorhabdus gen. nov. and Hydrogenovibrio, and reclassification of all four species of Thioalkalimicrobium to Thiomicrospira. Int J Syst Evol Micr 67:1140–1151.

10. Kojima H, Mochizuki J, Kanda M, Watanabe T, Fukui M. 2022. Thiomicrorhabdus immobilis sp. nov., a mesophilic sulfur-oxidizing bacterium isolated from sediment of a brackish lake in northern Japan. Arch Microbiol 204:605.

11. Liu X, Jiang L, Hu Q, Lyu J, Shao Z. 2020. Thiomicrorhabdus indica sp. nov., an obligately chemolithoautotrophic, sulfur-oxidizing bacterium isolated from a deep-sea hydrothermal vent environment. Int J Syst Evol Micr 70:234–239.

12. Gao Y, Zhu H, Wang J, Shao Z, Wei S, Wang R, Cheng R, Jiang L. 2023. Physiological and Genomic Characterization of a Novel Obligately Chemolithoautotrophic, Sulfur-Oxidizing Bacterium of Genus Thiomicrorhabdus Isolated from a Coastal Sediment. Microorganisms 11:2569.

13. Tan X-Y, Liu X-J, Li Z, Yu F, Yang H, Du Z-J, Ye M-Q. 2023. Thiomicrorhabdus marina sp.nov., an obligate chemolithoautotroph isolated from tidal zone sediment, and genome insight into the genus Thiomicrorhabdus. Frontiers Mar Sci 10:1144912.

14. Liu X, Chen B, Lai Q, Shao Z, Jiang L. 2021. Thiomicrorhabdus sediminis sp. nov. and Thiomicrorhabdus xiamenensis sp. nov., novel sulfur-oxidizing bacteria isolated from coastal sediments and an emended description of the genus Thiomicrorhabdus. Int J Syst Evol Micr 71.

15. Mochizuki J, Kojima H, Fukui M. 2021. Thiosulfativibrio zosterae gen. nov., sp. nov., and Thiosulfatimonas sediminis gen. nov., sp. nov. Arch Microbiol 203:951–957.

16. Zhou Z, John ESt, Anantharaman K, Reysenbach A-L. 2022. Global patterns of diversity and metabolism of microbial communities in deep-sea hydrothermal vent deposits. Microbiome 10:241.

17. Flores GE, Shakya M, Meneghin J, Yang ZK, Seewald JS, Wheat CG, Podar M, Reysenbach A‐L. 2012. Inter‐field variability in the microbial communities of hydrothermal vent deposits from a back‐arc basin. Geobiology 10:333–346.

18. Wang Y, Bi H-Y, Chen H-G, Zheng P-F, Zhou Y-L, Li J-T. 2022. Metagenomics Reveals Dominant Unusual Sulfur Oxidizers Inhabiting Active Hydrothermal Chimneys From the Southwest Indian Ridge. Front Microbiol 13:861795.

19. Brinkhoff T, Sievert SM, Kuever J, Muyzer G. 1999. Distribution and Diversity of Sulfur-Oxidizing Thiomicrospira spp. at a Shallow-Water Hydrothermal Vent in the Aegean Sea (Milos, Greece). Appl Environ Microb 65:3843–3849.

20. Magnuson E, Mykytczuk NCS, Pellerin A, Goordial J, Twine SM, Wing B, Foote SJ, Fulton K, Whyte LG. 2021. Thiomicrorhabdus streamers and sulfur cycling in perennial hypersaline cold springs in the Canadian high Arctic. Environ Microbiol 23:3384–3400.

21. Reysenbach A-L, John ESt, Meneghin J, Flores GE, Podar M, Dombrowski N, Spang A, L’Haridon S, Humphris SE, Ronde CEJ de, Tontini FC, Tivey M, Stucker VK, Stewart LC, Diehl A, Bach W. 2020. Complex subsurface hydrothermal fluid mixing at a submarine arc volcano supports distinct and highly diverse microbial communities. Proc National Acad Sci 117:32627–32638.

22. Zhou Z, Tran PQ, Kieft K, Anantharaman K. 2020. Genome diversification in globally distributed novel marine Proteobacteria is linked to environmental adaptation. Isme J 14:2060–2077.

23. Scott KM, Williams J, Porter CMB, Russel S, Harmer TL, Paul JH, Antonen KM, Bridges MK, Camper GJ, Campla CK, Casella LG, Chase E, Conrad JW, Cruz MC, Dunlap DS, Duran L, Fahsbender EM, Goldsmith DB, Keeley RF, Kondoff MR, Kussy BI, Lane MK, Lawler S, Leigh BA, Lewis C, Lostal LM, Marking D, Mancera PA, McClenthan EC, McIntyre EA, Mine JA, Modi S, Moore BD, Morgan WA, Nelson KM, Nguyen KN, Ogburn N, Parrino DG, Pedapudi AD, Pelham RP, Preece AM, Rampersad EA, Richardson JC, Rodgers CM, Schaffer BL, Sheridan NE, Solone MR, Staley ZR, Tabuchi M, Waide RJ, Wanjugi PW, Young S, Clum A, Daum C, Huntemann M, Ivanova N, Kyrpides N, Mikhailova N, Palaniappan K, Pillay M, Reddy TBK, Shapiro N, Stamatis D, Varghese N, Woyke T, Boden R, Freyermuth SK, Kerfeld CA. 2018. Genomes of ubiquitous marine and hypersaline Hydrogenovibrio, Thiomicrorhabdus and Thiomicrospira spp. encode a diversity of mechanisms to sustain chemolithoautotrophy in heterogeneous environments. Environ Microbiol 20:2686–2708.

24. Galmés J, Kapralov MV, Copolovici LO, Hermida-Carrera C, Niinemets Ü. 2015. Temperature responses of the Rubisco maximum carboxylase activity across domains of life: phylogenetic signals, trade-offs, and importance for carbon gain. Photosynth Res 123:183–201.

25. Galmés J, Hermida-Carrera C, Laanisto L, Niinemets Ü. 2016. A compendium of temperature responses of Rubisco kinetic traits: variability among and within photosynthetic groups and impacts on photosynthesis modeling. J Exp Bot 67:5067–5091.

26. Hayashi NR, Oguni A, Yaguchi T, Chung S-Y, Nishihara H, Kodama T, Igarashi Y. 1998. Different properties of gene products of three sets ribulose 1,5-bisphosphate carboxylase/oxygenase from a marine obligately autotrophic hydrogen-oxidizing bacterium, Hydrogenovibrio marinus strain MH-110. J Ferment Bioeng 85:150–155.

27. Davidi D, Shamshoum M, Guo Z, Bar‐On YM, Prywes N, Oz A, Jablonska J, Flamholz A, Wernick DG, Antonovsky N, Pins B de, Shachar L, Hochhauser D, Peleg Y, Albeck S, Sharon I, Mueller‐Cajar O, Milo R. 2020. Highly active rubiscos discovered by systematic interrogation of natural sequence diversity. Embo J 39:e104081.

28. Flamholz AI, Prywes N, Moran U, Davidi D, Bar-On YM, Oltrogge LM, Alves R, Savage D, Milo R. 2019. Revisiting Trade-offs between Rubisco Kinetic Parameters. Biochemistry 58:3365–3376.

29. Rapp JZ, Sullivan MB, Deming JW. 2021. Divergent Genomic Adaptations in the Microbiomes of Arctic Subzero Sea-Ice and Cryopeg Brines. Frontiers in Microbiology 12.

30. Raymond-Bouchard I, Goordial J, Zolotarov Y, Ronholm J, Stromvik M, Bakermans C, Whyte LG. 2018. Conserved genomic and amino acid traits of cold adaptation in subzero-growing Arctic permafrost bacteria. Fems Microbiol Ecol 94.
